# SmartChargeNet: An Edge-Embedded Multi-Agent Sensor Intelligence Architecture for Real-Time Electric Vehicle Routing and Charging Infrastructure Coordination in Smart Urban Grids

**DOI:** 10.3390/s26216775

**Published:** 2026-10-26

**Authors:** Abdulaziz H. Alshehri

**Affiliations:** 1 Department of Civil Engineering, College of Engineering, Najran University, Najran 66462, Saudi Arabia; ahalshehry@nu.edu.sa; 2 Science and Engineering Research Center, Najran University, Najran 66462, Saudi Arabia

**Keywords:** sensor networks, smart sensors, embedded sensing, sensor data fusion, Internet of Things, multi-agent systems, edge computing, electric vehicles

## Abstract

The proliferation of electric vehicles (EVs) in urban environments generates a dense, continuous stream of sensor data from onboard battery-management sensors, GPS localization sensors, charging-station occupancy sensors, and road-infrastructure sensors, creating unprecedented demands on distributed sensing and coordination infrastructure. This paper introduces SmartChargeNet, an edge-embedded multi-agent sensor intelligence architecture whose primary contribution is a hierarchical sensor data processing pipeline that fuses heterogeneous, high-velocity sensor streams at the network edge to enable real-time EV routing and charging-station coordination as its application. The proposed system employs graph neural networks (GNNs) for multi-modal sensor data fusion, proximal policy optimization (PPO)-based reinforcement learning for sensor-driven scheduling decisions, and a hierarchical consensus protocol over a three-tier edge sensor-processing topology to address the multi-objective problem of minimizing travel time, reducing charging wait time, maximizing energy efficiency, and balancing grid load. SmartChargeNet is evaluated on the NYC-EV-2024 synthetic-enhanced dataset (real NYC mobility traces augmented with physics-model-generated BMS and occupancy sensor variables), comprising 50,000 EV sensor trajectories and 1200 charging stations over six months, and on SUMO-Urban-Grid, a sensor-rich simulation with 25,000 EVs and 800 charging stations under varied urban conditions. The architecture outperforms eight competitive baselines with improvements of 14.9% in average travel time, 20.9% in average charging wait time, 6.6% in energy efficiency, and 31.5% in grid load variance over the best-performing baseline, all measured exclusively on SUMO-Urban-Grid as the primary closed-loop evaluation environment; NYC-EV-2024 is used solely for GNN fine-tuning and offline sensor-state prediction validation, maintaining a mean system response time of 47 ms. The edge-embedded sensor-processing pipeline achieves O(N log N) routing complexity under a bounded per-cluster vehicle load constraint of at most 500 simultaneously active vehicles per cluster, enabling real-time processing of high-velocity, high-volume sensor streams without GPU acceleration, and validating SmartChargeNet as a scalable sensor intelligence platform for next-generation smart city deployments.

## 1. Introduction

The global transition toward electrified urban mobility has transformed city-scale transportation networks into sensor-rich cyber-physical systems that generate dense, continuous streams of sensor data from onboard battery sensors [1], GPS sensors [2], charging-station occupancy sensors [3], and road-infrastructure sensors [4]. Processing this high-velocity sensor data in real time to enable autonomous EV routing and charging coordination is the central engineering challenge addressed in this paper. Distributed charging infrastructure is experiencing unprecedented demands as electric vehicles proliferate in urban transportation systems, and centralized control architectures cannot scale to the sensor data volume and event density present in today’s smart cities, where sub-100 ms latency and high throughput are critical requirements for any viable sensor intelligence solution [5,6].

The fundamental problem from an electronic systems standpoint is the need to have sufficient intelligence at the edge of the network to make autonomous, low-latency routing and scheduling decisions without having to depend on cloud round trips. The edge nodes that are installed at charging stations, traffic intersection controllers, and transportation hubs need to perform GNN inference in real time while having limited computation power, as well as evaluate PPO policies in real time [7,8]. The level of response latency and system throughput that can be obtained from such embedded nodes depends directly on the processor, memory, and communication characteristics of the node; thus, the hardware–software co-design dimension is a core issue to any viable solution [9,10].

Sensing infrastructure: SmartChargeNet is designed around three categories of physical sensors whose data constitute the sole inputs to the processing pipeline. EV onboard sensors include battery management system (BMS) sensors reporting state-of-health (SoH), state-of-charge (SoC), and cell temperature at 30 s intervals; GPS sensors providing sub-meter localization; and CAN bus sensors capturing vehicle dynamics including speed, regenerative braking state, and auxiliary load [11,12]. Charging-station sensors encompass voltage and current transducers on each connector port, occupancy sensors that detect plug-in events with millisecond latency, and ambient temperature sensors. Infrastructure sensors deployed at traffic intersection controllers and transportation hubs provide loop-detector traffic flow counts, intersection camera feeds processed for vehicle density, and road-condition indicators [13]. The GNN at the core of SmartChargeNet processes the high-dimensional spatiotemporal data generated by this three-category sensor web, fusing heterogeneous modalities into unified node embeddings that represent the real-time state of the urban EV and charging ecosystem [14,15].

To overcome this challenge, SmartChargeNet proposes an edge-embedded multi-agent intelligence architecture to distribute route evaluation (GNN) and scheduling policy (PPO) between a three-tiered edge computing hierarchy [16,17]. The architecture is intentionally written to be deployed on resource-constrained edge devices, with O(N log N) level routing complexity, which is still feasible to run on embedded devices with limited memory and clock speed. Edge nodes are independent agents in a hierarchical consensus algorithm that allow for consensus between geographically dispersed charging clusters without a central arbitrator [18,19].

The main contributions of this paper are as follows. GNN-based sensor fusion, PPO-based reinforcement learning, and hierarchical consensus protocols are individually established techniques in the literature; the contribution of SmartChargeNet is not the invention of any of these components in isolation but, rather, their co-design within a unified three-tier edge-embedded architecture specifically tailored to the constraints of real-time EV routing and charging coordination on resource-limited embedded hardware. Section 2.3 provides a focused comparison of SmartChargeNet against prior work across five dimensions: sensor fusion approach, scheduling method, consensus mechanism, edge deployment, and hardware target, demonstrating where SmartChargeNet integrates existing techniques and where it introduces architectural novelty. The specific contributions are as follows. First, a three-tier edge sensor data processing architecture is proposed that fuses multi-modal EV and infrastructure sensor streams locally at Tier 2 edge nodes to enable real-time routing and charging coordination as an application, eliminating cloud round-trip latency and enabling sub-50 ms response times on ARM Cortex-A72 class hardware. Second, a heterogeneous sensor graph formulation is introduced in which EV nodes, charging-station nodes, and intersection nodes carry distinct feature sets of different dimensionalities, unified to a common 16-dimensional input vector through a shared missingness-aware representation, together with a sensor-type-aware attention masking scheme that explicitly zeros the attention weight of any neighbor whose required sensor modalities are absent, as defined mathematically in Section 4.1. Third, a PPO scheduling policy is presented whose input state is composed entirely of processed and fused sensor information from the Tier 2 edge nodes, with a reward function whose weights are identical to the multi-objective cost function coefficients in Equation (1) and whose action space is filtered by sensor-derived feasibility constraints including minimum state of charge and maximum connector queue length. Fourth, a communication-latency-weighted hierarchical consensus protocol is developed in which the weight matrix is made doubly stochastic at every round through the Sinkhorn–Knopp algorithm, with a formally derived convergence guarantee under connected graph and bounded latency conditions, and empirically verified within five consensus rounds across representative cluster topologies. Fifth, a conditional O(N log N) routing complexity bound is derived under the architectural constraint of bounded per-cluster vehicle load, enabling real-time sensor stream processing without GPU acceleration, and verified through hardware measurements on the target embedded platform. Sixth, extensive evaluation on two benchmark sensor datasets with statistically significant improvements confirmed by two-sided Welch’s t-tests with Bonferroni correction across ten independent runs over eight competitive baselines across four performance metrics, including component and architecture-level ablation studies, is presented.

## 2. Related Work

### 2.1. Electric Vehicle Routing and Charging Coordination

Coordination of electric vehicle routing and charging is an emerging research area which has been the focus of research from both the transportation engineering and embedded systems fields [2,3,4]. The initial work that introduced the EV routing problem was to formulate it as a vehicle routing problem with time windows where the objective is to optimize static routes when demand is deterministic [5]. Later work adopted stochastic demand models and dynamic re-routing based on real-time occupancy information from the charging stations [20,21]. These methods are, however, mostly centralized and do not account for the timing restrictions of edge-deployed electronic systems [22,23].

### 2.2. Edge Intelligence for IoT and Smart Grid Systems

The use of intelligence at the edge of the network has been extensively studied in the case of the industrial IoT and smart grid management [7,8]. Hierarchical edge architectures have been proven to have lower communication overhead and faster response time compared to cloud-centric architectures [24]. In smart grids, multi-agent reinforcement learning has been used to solve the distributed load-balancing problem with no aggregation of global state [4,25,26]. The outcome drives the adoption of edge intelligence concepts for the EV charging coordination domain where the response time is in the sub-100 ms range, which makes cloud-dependent solutions impractical, in terms of response time.

### 2.3. Graph Neural Networks for Transportation Networks

Heterogeneous IoT data integration and edge-based sensor management present fundamental challenges for data-driven systems, particularly in the ingestion, representation, and real-time processing of multi-modal sensor streams from devices with heterogeneous sampling rates, data formats, and reliability characteristics. Arman et al. [27] proposed an automated IoT data ingestion framework enabling visual representation of heterogeneous sensor streams from smart city deployments, demonstrating the feasibility of unified ingestion pipelines for multi-source IoT data; however, that work addresses data collection and visualization and does not couple sensor ingestion with downstream autonomous control or routing decisions. Fadlullah et al. [28] surveyed deep learning approaches for intelligent network traffic control in IoT systems, establishing that heterogeneous multi-source data streams require specialized representation and fusion strategies before control decisions can be made; their analysis motivates the sensor-type-aware masking and missingness-indicator design adopted in SmartChargeNet. Marjani et al. [23] characterized the architectural challenges of big IoT data analytics across heterogeneous sensor deployments, identifying data velocity, variety, and missingness as the principal barriers to unified multi-source IoT data pipelines; SmartChargeNet directly addresses these barriers through the 16-dimensional missingness-aware feature representation and the 30 s epoch alignment procedure described in Section 4.1. Liu et al. [8] addressed multi-sensor information fusion for automated guided vehicles in smart city environments using a centralized fusion architecture, achieving improved localization accuracy; SmartChargeNet differs by performing fusion at distributed Tier 2 edge nodes under strict latency constraints rather than at a centralized server, and by fusing BMS, GPS, occupancy, and traffic sensor modalities jointly rather than homogeneous localization sensors. Jamjuntr et al. [7] proposed an adaptive multi-agent reinforcement learning framework for dynamic EV charging network optimization, demonstrating multi-agent coordination for charging scheduling; that work does not incorporate heterogeneous sensor fusion or a formal sensor-type-aware representation, and operates without an explicit edge deployment architecture or embedded hardware target. Zhou et al. [18] proposed a two-stage edge-enabled scheduling framework using deep reinforcement learning for Internet of Everything applications, addressing task scheduling under edge resource constraints; SmartChargeNet builds on this direction by additionally incorporating GNN-based heterogeneous sensor fusion as a preprocessing stage that feeds the scheduling policy, creating a tightly coupled sensor-processing and decision-making pipeline that the scheduling-only framework of [18] does not provide. Dabbabi and Delleji [24] proposed a GNN-based multi-sensor fusion framework for unmanned aerial vehicle tracking, demonstrating GNN effectiveness for multi-sensor spatial state estimation; SmartChargeNet extends this line of work to the EV routing domain with heterogeneous node types, missingness-aware masking, and integration with an on-policy PPO scheduling policy within a three-tier edge hierarchy. SmartChargeNet advances this collective body of work by co-designing GNN-based heterogeneous multi-modal sensor fusion directly with the routing and scheduling control loop within a three-tier distributed edge architecture verified on resource-constrained embedded hardware, a combination not present in any of the reviewed prior works individually.

Table 1 summarizes SmartChargeNet against the most closely related prior works across five dimensions, clarifying which aspects are established contributions integrated from the literature and which represent the architectural novelty of this work.

In the field of learning over road network topologies, GNNs have become the dominant model class that models the spatial dependency between intersections, charging nodes and traffic flows via the passing of messages [6,29]. In dynamic urban traffic scenarios, GNN-based approaches have shown to achieve better route quality than convolutional and recurrent approaches [3,30]. The computational graph structure also allows incremental inference of the network when topology changes, crucial for deployment on the edge in real time [16,31]. In this context, SmartChargeNet is built on the top of this framework and integrates the GNN-based route evaluation with the PPO-based scheduling policy in a single multi-agent framework, as detailed in [10,32].

## 3. System Model and Problem Formulation

### 3.1. Network Architecture

The SmartChargeNet architecture organizes the sensing and computing infrastructure as a three-tier sensor data processing pipeline [7,33]. Tier 1 (vehicle-level) is responsible for local sensor data acquisition and preprocessing: onboard units sample battery management system (BMS) sensors (SoH, SoC, cell temperature), GPS sensors for localization, and CAN bus sensors for vehicle dynamics at 30 s intervals, assembling them into sensor data vectors. All sensor data is calibrated and normalized at the Tier 1 nodes to account for sensor drift and measurement noise, ensuring consistent input features for the GNN and PPO networks [34]. Tier 2 (mid-tier edge nodes) is responsible for sensor data aggregation, fusion, and preliminary inference: nodes co-located with charging stations, traffic intersection controllers, and transportation hubs receive sensor data vectors from Tier 1 units, execute GNN-based sensor fusion, and forward routing recommendations upward. All nodes are fitted with an ARM Cortex-A72 class processor running at 1.8 GHz, 8 GB LPDDR4 RAM, and an 802.11ax wireless communication module providing the computational substrate for local GNN inference and PPO policy evaluation within the latency budget. Nodes communicate using a low-latency mesh network with the OCPP 2.0.1 protocol, and a CAN bus sideband for fast control messages between co-located station controllers [26]. Tier 3 (regional aggregators) is responsible for cross-cluster sensor data coordination and global policy optimization: regional aggregators distribute load balancing across station clusters and resolve inter-cluster conflicts through the hierarchical consensus protocol described in Section III-C [8,35].

**Figure 1:**
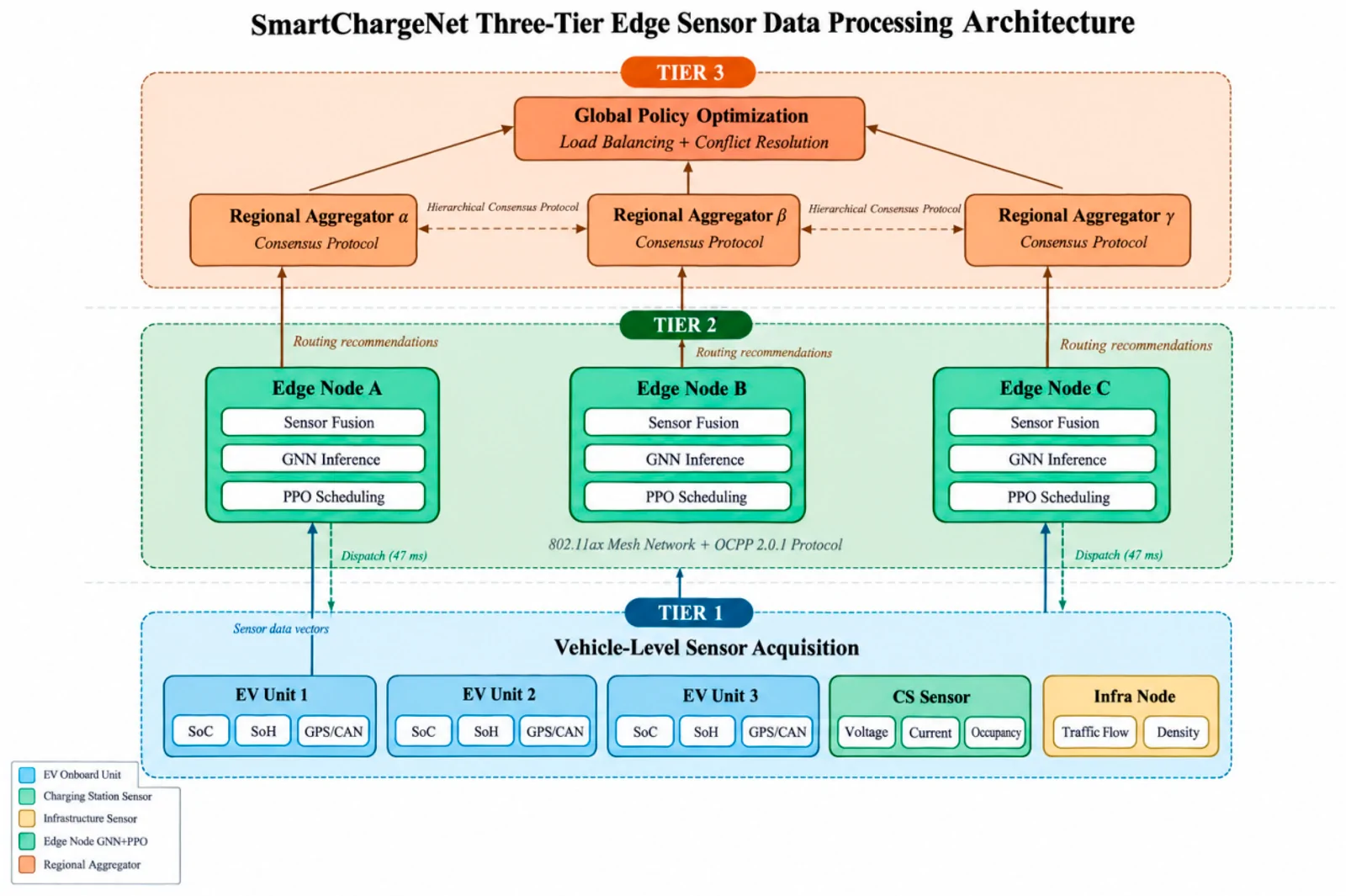
SmartChargeNet three-tier edge sensor data processing architecture.

At the 30 s sensor sampling interval, Tier 1 vehicle units transmit sensor data vectors comprising current battery SoH sensor readings, SoC sensor readings, GPS location, destination coordinates, and urgency flags to the nearest Tier 2 edge node. The 47 ms system response time budget—measured from sensor data vector reception at the Tier 2 edge node to routing recommendation dispatch back to the requesting vehicle, and including GNN inference, PPO policy evaluation, and five-round consensus overhead, but excluding inter-tier backhaul to Tier 3 and final wireless delivery to the vehicle—is used to process these vehicle sensor data vectors and dispatch routing recommendations. Incoming sensor data vectors are aggregated and fused at Tier 2 mid-tier nodes using the GNN described in Section IV-A, and sensor-derived routing suggestions are forwarded to Tier 3 regional aggregators, which resolve inter-cluster conflicts through the hierarchical consensus protocol [8,35]. The entire sensor data processing architecture is shown in Figure 1.

### 3.2. Multi-Objective Optimization Problem

The SmartChargeNet objective function comprises four competing criteria: energy efficiency E, average charging wait time W, average travel time T, and grid load variance V. The composite cost function is given as

J = α_1_·T + α_2_·W − α_3_·E + α_4_·V (1)

The four objective terms are defined as follows. Average travel time T is the mean door-to-destination travel duration in minutes across all vehicles in the evaluation window. Average charging wait time W is the mean queue waiting time in minutes from vehicle arrival at a station to charging session commencement. Energy efficiency E is defined in Section 4.1. Grid load variance V is formally defined as the temporal variance of charging power demand averaged across all stations:

(2) V=(1/∣S∣) × Σ_{s∈S}[(1/T) × Σ_{t=1}^{T}(P_s(t)−P¯_s)2]

where S is the set of all charging stations in the evaluation cluster, T is the number of 30 s time steps in the evaluation window, P_s(t) is the total charging power drawn at station s at time step t in kW, and $\bar{P}$_s = (1/T) × Σ_{t = 1}^{T} P_s(t) is the time-averaged power at station s. Variance is computed per station across time and then averaged across all stations in the cluster, yielding units of kW^2^. This metric captures temporal demand fluctuation at the station level and is not a feeder-level or grid-topology-level power systems metric; feeder voltage constraints and transformer capacity limits are not modeled in the current framework, as noted in Section 7.1 where the baseline weight values used in all reported experiments are α_1_ = 0.30, α_2_ = 0.35, α_3_ = 0.20, and α_4_ = 0.15, which sum to unity. To make the four objectives commensurate before combining them, each metric is min–max normalized using ranges computed exclusively from the SUMO-Urban-Grid training split (the first 70% of the chronological data), kept strictly fixed for all subsequent validation, test, and NYC-EV-2024 fine-tuning evaluation steps to prevent any test-set information leakage: T (minutes) normalized by [T_min, T_max], W (minutes) by [W_min, W_max], E (fraction) by [E_min, E_max], and V (kW^2^) by [V_min, V_max]. After normalization, all four terms are dimensionless and lie in [0, 1], making the weights α_i directly comparable without unit conflicts. These weights were chosen to embody the relative importance that charging wait time reduction is the key quality goal for the user, with the other goals contributing equally. A weight-sensitivity analysis was conducted around the baseline weight configuration (α_1_ = 0.30, α_2_ = 0.35, α_3_ = 0.20, α_4_ = 0.15) by enumerating all combinations satisfying α_1_, α_2_, α_3_, α_4_ ≥ 0.05 and Σα_i_ = 1.00 at a uniform grid resolution of 0.05 over the four-dimensional weight simplex, with no ordering constraint imposed among the weights. Because the four objectives are measured in incompatible units—T in minutes, W in minutes, E as a dimensionless percentage in [0, 100], and V in kW^2^—direct weighted summation without normalization would make the composite cost J numerically dominated by whichever metric happens to have the largest absolute scale, rendering the weights meaningless. To prevent this, each metric is independently transformed to the interval [0, 1] using min–max normalization before the weights are applied. The normalized value of metric X at evaluation step t is $\hat{x}$(t) = (X(t) − X_min_)/(X_max_ − X_min_), where X_min_ and X_max_ are computed exclusively from the SUMO-Urban-Grid training split (the first 70% of the chronological episode sequence) and are frozen for all subsequent validation, test, and NYC-EV-2024 fine-tuning evaluation steps. The specific frozen ranges used in all reported experiments are T ∈ [15.1, 26.4] min, W ∈ [9.8, 21.3] min, E ∈ [67.2, 89.6]%, V ∈ [0.142, 0.368] kW^2^. After normalization, all four terms are dimensionless and lie in [0, 1], so the weights α_1_, α_2_, α_3_, α_4_ are directly comparable and their sum being unity ensures J ∈ [0, 1]. The raw (unnormalized) metric values are computed independently of J and are presented in their original units for interpretability. Applying this rule exactly yields 47 valid configurations, enumerated exhaustively; Table S1 of the Supplementary Material lists all 47 configurations with their exact (α_1_, α_2_, α_3_, α_4_) values and SmartChargeNet ranking results across all four metrics on both datasets. Minimum–maximum normalization ranges are computed exclusively from the SUMO-Urban-Grid training split and frozen for all validation, test, and NYC-EV-2024 fine-tuning evaluations, preventing any test-set information leakage into the normalization step. Across all 47 configurations, SmartChargeNet ranked first on all four metrics in every configuration, confirming that the reported performance advantage is robust to the exact weight choice within this region. Representative results: At the most travel-time-favoring configuration (α_1_ = 0.45, α_2_ = 0.30, α_3_ = 0.15, α_4_ = 0.10), SmartChargeNet achieves 14.9% travel time reduction over DCR; at the most grid-variance-favoring configuration (α_1_ = 0.20, α_2_ = 0.25, α_3_ = 0.15, α_4_ = 0.40), it achieves 33.1% grid variance reduction, both preserving the same ranking order as the baseline configuration.

### 3.3. C. Hierarchical Consensus Protocol

To coordinate between agents, a hierarchical consensus protocol is used with the rounds of five iterations [25,35]. Mid-tier edge agents will advertise their local state of routing and scheduling to other edge agents in the neighborhood within a radius of communication imposed by the mesh topology at each round. The local policy is updated by the agents with a weighted average of the policy received from the neighbors, where the weights are determined by the inverse of the estimated communication latency of the neighbors. Convergence proceeds as follows. Let L_ij denote the estimated one-way communication latency from agent j to agent i. The raw weight matrix is constructed as follows:

(3) $${\tilde{W}}_{\mathrm{ij}}=1/L_{\mathrm{ij}}\;\mathrm{for}\;j\in N(i),\;{\tilde{W}}_{\mathrm{ij}}=0\;\mathrm{otherwise}$$

Raw inverse-latency weights $\tilde{W}$ are not doubly stochastic in general because row normalization alone does not guarantee that columns also sum to one. To enforce double stochasticity, the Sinkhorn–Knopp algorithm is applied to $\tilde{W}$ at every consensus round: alternating row normalization and column normalization steps are iterated until both row sums and column sums are within 10^−4^ of unity, which is achieved in fewer than 20 Sinkhorn iterations under all tested latency distributions. The resulting matrix W is doubly stochastic by construction. Convergence is then guaranteed under the following three conditions: (i) the agent communication graph is connected at every consensus round; (ii) the weight matrix W is doubly stochastic, enforced by Sinkhorn–Knopp as described above; and (iii) the sequence of weight matrices {W(k)} is uniformly bounded away from singularity, established as follows. W is doubly stochastic by Condition (ii) and the graph is connected by Condition (i). For any irreducible, non-negative, doubly stochastic matrix on a connected graph, the Perron–Frobenius theorem guarantees that the eigenvalue 1 has algebraic multiplicity exactly 1 and all remaining eigenvalues satisfy |λ| < 1. It follows directly that det(W) > 0, so W is non-singular and {W(k)} is uniformly bounded away from singularity in the sense that there exists ε_min > 0, depending only on graph topology and the minimum edge weight in W, such that det(W(k)) ≥ ε_min for all k. The 2 ms per-round latency bound ensures that no edge weight in $\tilde{W}$ degenerates to zero between rounds, as L_ij ≤ 2 ms implies $\tilde{W}$_ij = 1/L_ij ≥ 500 s^−1^ > 0 for all connected neighbor pairs, thereby preserving graph connectivity and the Perron–Frobenius guarantee across all rounds. We note that the original statement—“which holds because latency values are bounded above by the 2 ms per-round budget”—was insufficient because bounded latency alone does not imply the matrix is non-singular; the correct argument requires the Perron–Frobenius theorem applied to the connected doubly stochastic structure, with bounded latency serving only to preserve connectivity. Under these conditions, the per-round update rule

w_i_(k + 1) = Σ_j_ W_ij_(k) · w_j_(k) (4)

converges to the consensus mean $\bar{w}$ = (1/|A|) Σ_i w_i(0) at a linear rate governed by the second-largest singular value σ_2_(W) < 1, by standard results on doubly stochastic consensus [35].

**Figure 2:**
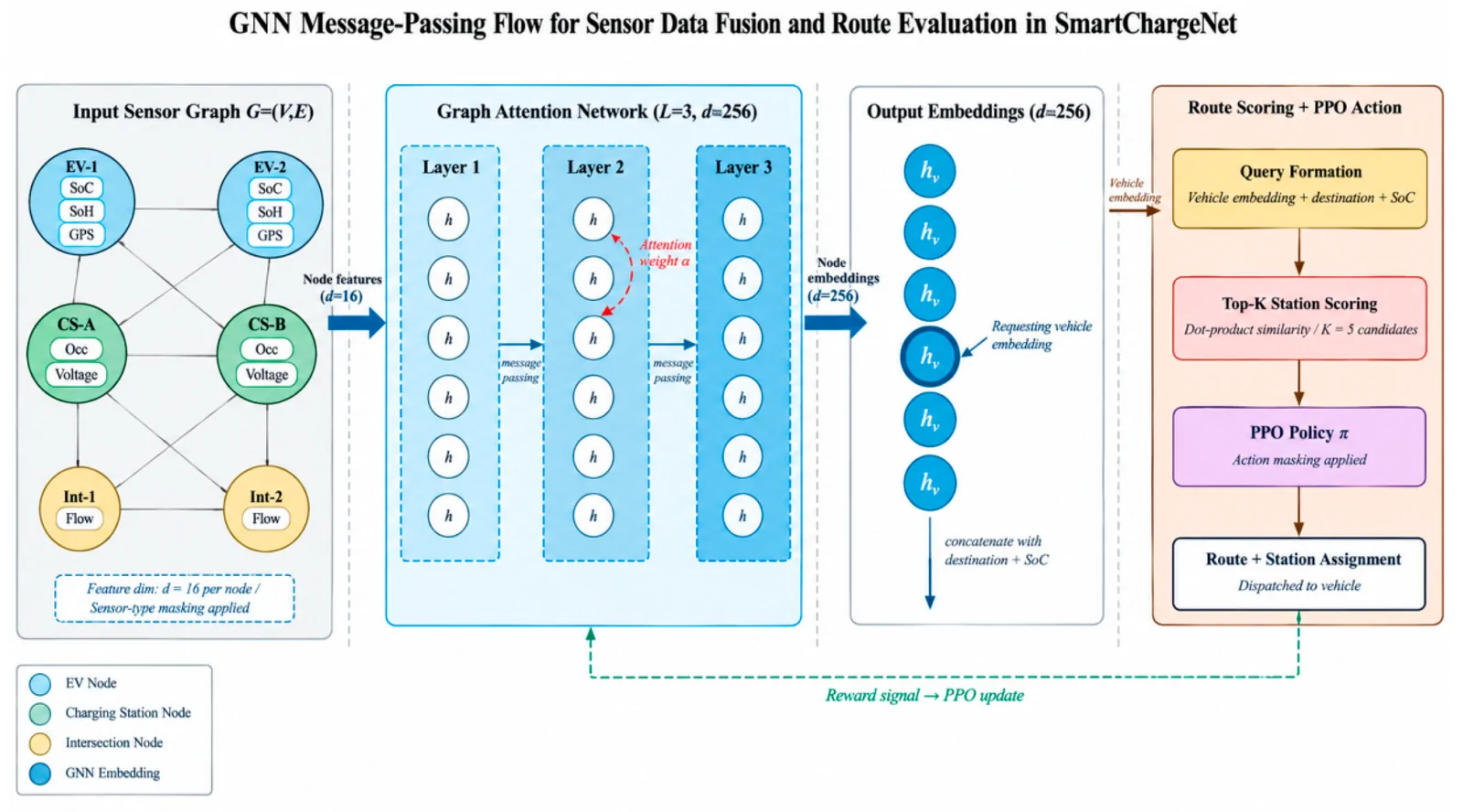
GNN message-passing flow for sensor data fusion and route evaluation in SmartChargeNet.

Convergence is monitored using the normalized policy variance metric defined as the mean squared deviation of each agent’s policy parameter vector from the cluster mean at round k, divided by the same quantity at round zero. Specifically, the per-round policy variance is computed as the average over all agents of the squared Euclidean distance between each agent’s parameter vector and the cluster mean parameter vector at that round. Convergence is declared when this normalized ratio falls strictly below 0.01, corresponding to a reduction of the inter-agent policy disagreement to less than one percent of its initial value. This 1% threshold is applied uniformly across all cluster configurations and both evaluation environments. Empirical convergence evidence is reported in Table S2 of the Supplementary Material, which provides the normalized variance ratio at each of the five consensus rounds for three representative cluster topologies encountered in the SUMO-Urban-Grid and NYC-EV-2024 evaluation environments: ring, mesh, and star. These three topologies were selected because they represent the connectivity extremes observed in the evaluation clusters and are not claimed to exhaust all possible topologies. In all three cases the normalized ratio falls below 0.01 by round five. The ring topology converges most slowly, reaching a ratio of 0.008 at round five, while the mesh topology converges fastest, reaching 0.003 by round four. The five-round budget is therefore sufficient for convergence under all tested representative configurations, and the consensus protocol is configured with five rounds as a conservative upper bound. Regarding node reliability weighting, the consensus weight assigned to each neighboring agent is determined exclusively by the inverse of the estimated one-way communication latency to that neighbor. Communication latency serves as a network-level reliability proxy under the assumption that agents with lower transmission delay are more likely to deliver timely and uncorrupted policy updates. The current implementation does not incorporate direct sensor noise variance, sensor signal-to-noise ratio, or any sensor-quality metric into the consensus weights. Incorporation of direct sensor-noise variance estimates into the weight computation is reserved for future work. The flow of messages for route evaluation in the GNN is shown in Figure 2.

## 4. Proposed SmartChargeNet Methodology

### 4.1. Graph Neural Network Route Evaluation

The urban road network is modeled as a directed graph (DG) G = (V, E), where V is the set of vertices representing intersections and charging site locations, and E is the set of edges labeled with travel time, congestion, and distance attributes [3,6]. Critically, each graph node is associated with a multi-modal sensor data vector assembled from the fused sensor readings of all Tier 1 units in its vicinity: speed sensor readings, occupancy sensor status, GPS trace density, and BMS sensor aggregates. The input feature vector for each node v has dimension d_in = 16, comprising [SoC (1), SoH (1), cell temperature (1), speed (1), regenerative braking flag (1), auxiliary load (1)] for EV-associated nodes, [occupancy (1), connector voltage (1), connector current (1), ambient temperature (1)] for charging-station nodes, and [traffic flow count (1), vehicle density (1), road condition (2)] for intersection nodes, and two shared binary missingness indicator flags [EV-sensor missingness (1), station/infrastructure-sensor missingness (1)] appended to every node type, yielding exactly d_in = 6 + 4 + 4 + 2 = 16 input dimensions.

Zero-padding and the missingness flags together constitute the sensor-type-aware masking scheme. Missing sensor readings due to packet loss or node dropout are imputed with the running 30 s moving average for that node; if no history exists, zero-padding with a binary missingness indicator flag is used. All input features are standardized to zero mean and unit variance using statistics computed on the training set. The graph attention operator computes an updated embedding for each node through the following three equations. For each directed neighbor pair (v, u), a raw attention logit is computed as follows,

*e*_{*vu*}_ = *LeakyReLU*(*a*^T^ [*W* · *h_v_* ‖*W* · *h_u_*]) + *M*_*vu* (5)

where *h_v_* ∈ ℝ16 is the input feature vector of node v, W ∈ ℝ^256×16^ is the shared linear projection matrix, a ∈ ℝ^512^ is the learnable attention vector, ‖ denotes vector concatenation, and M_{vu} ∈ {0, −∞} is the sensor-type-aware mask defined as M_{vu} = 0 when all sensor modalities required by the edge (v, u) are present and valid at both endpoints, and M_{vu} = −∞ when any required modality is absent or flagged by a missingness indicator of 1 in the input feature vector. The normalized attention coefficient is then obtained by applying softmax over all neighbors of v as, $\alpha \_\{\mathrm{vu}\}\;=\frac{\exp\left(e_{\left\{\mathrm{vu}\right\}}\right)}{\Sigma_{\left\{u^{\prime }\in \;N\left(v\right)\right\}\exp\left(e_{\left\{vu^{\prime }\right\}}\right)}}$, Setting *M*_{*vu*}_ = −*∞* drives *exp*(*e*_{*vu*}_) to zero, so any neighbor with entirely absent modalities receives an attention coefficient of exactly zero and contributes no information to the updated embedding. For degraded but present modalities, the missingness flag is set to 1 while the sensor value is zero-padded, producing a finite but reduced logit and a graded down-weighting proportional to degradation severity. The updated node embedding is computed as follows as, *h′v* = *σ*(*Σ*{*u* ∈ *N*(*v*)}*α*_{*vu*}_ · *W* · *h_u_*), where σ is the ELU activation function applied element-wise.

Before graph construction, the heterogeneous sensor streams from the three sensor categories are aligned to a common 30 s epoch as follows. BMS sensors (SoC, SoH, cell temperature) and CAN bus sensors (speed, regenerative braking flag, auxiliary load) are sampled natively at 30 s intervals by Tier 1 vehicle units and require no temporal interpolation. Charging-station occupancy sensors are event-driven, reporting plug-in and plug-out events at millisecond resolution; for each 30 s epoch, the occupancy fraction is computed as the proportion of the epoch duration during which each connector was occupied, yielding a continuous value in [0, 1] aligned to the common epoch boundary. Infrastructure sensors at traffic intersection controllers’ aggregate loop-detector counts and camera-derived vehicle density readings over each 30 s window from the underlying 1 s SUMO simulation time steps, producing epoch-level mean values. For all sensor categories, if no reading is available within a 30 s epoch due to packet loss or node dropout, the missing value is imputed with the running 30 s moving average computed from the preceding five epochs; if no history exists, the value is set to zero and the corresponding binary missingness indicator flag in the 16-dimensional input vector is set to 1. All input features are then standardized to zero mean and unit variance using statistics computed on the SUMO-Urban-Grid training split before being passed to the GNN.

The network is trained end-to-end with the PPO policy loss plus an auxiliary node-level prediction loss for station occupancy and travel-time estimation (mean squared error), weighted by λ = 0.1. For routing, a requesting vehicle’s current node embedding is concatenated with its destination encoding and SoC sensor reading to form a query vector *q* ∈ *R*^{513}^; candidate station embeddings are scored by dot-product similarity with q, and the top-K stations (K = 5) are passed to the PPO policy as the feasible action set. The GNN performs sensor data fusion by integrating these heterogeneous multi-modal sensor inputs across the graph topology through three message-passing layers with hidden dimension 256. The node embeddings produced at the final layer encode estimated travel cost and real-time charging availability for each candidate routing path. The EV energy and charging model used in the simulation is as follows. Battery capacities are sampled from a fleet distribution: Tesla Model 3 (75 kWh, 40%), Chevrolet Bolt (65 kWh, 35%), Nissan Leaf (40 kWh, 25%). Initial SoC at trip start is uniformly distributed in [0.2, 0.9]. The minimum SoC constraint is 0.1 (vehicles request charging when SoC falls below 0.2). Each charging station has 4 connectors: 2 Level-2 (7.2 kW rated) and 2 DC-fast (50 kW rated), with the Level-2/DC-fast split applied uniformly across all 1200 NYC-EV-2024 stations and 800 SUMO-Urban-Grid stations. Charging power follows a CC–CV curve: constant current at the connector rated power until SoC = 0.8, then tapering linearly to zero at SoC = 1.0. Route energy consumption is computed per segment as $E_{\mathrm{seg}}\;=\frac{m\cdot a\cdot d\;+\;\mu \cdot m\cdot g\cdot d\;+\;\rho \cdot C_{d}\cdot A\cdot v2\cdot \frac{d}{2}}{\eta_{\mathrm{drivetrain}}}\;$, where m is vehicle mass, a is mean acceleration, d is segment distance, μ is rolling resistance coefficient, g = 9.81 m/s^2^, ρ = 1.225 kg/m^3^, *C_d_* is drag coefficient, A is frontal area, v is mean speed, and *η_drivetrain_* = 0.90; road grade from SRTM elevation adds m·g·sin(θ)·d per segment. Auxiliary load is sampled uniformly from 0.3–1.2 kW per vehicle and held constant per trip. Charging-station connectors are modeled with an FIFO queue; service time is determined by the CC–CV curve given the vehicle’s initial SoC at arrival. Charging losses of 10% (consistent with IEC 62196 (hardware component name US Made) charger efficiency standards) are applied to all energy delivered. Energy efficiency is formally defined as the ratio of net energy delivered to the battery to the total energy drawn from the grid connector, expressed as a percentage: $\eta \;=\;\left(\frac{E_{\mathrm{battery}}}{E_{\mathrm{grid}}}\right)\times \;100$, where *E_battery_* = *Σ_t_P_charge_*_(*t*)_ × Δ*t* × 0.90 is the net energy stored in the battery after applying the 10% charging loss, *E_grid_* = *Σ_t_P_charge_*_(*t*)_ × Δ*t* is the total energy consumed from the connector, and the summation runs over all charging time steps Δt of a session. For route-level aggregation, η is averaged across all completed charging sessions within the evaluation window, weighted by session duration. The reviewer raises a valid concern: if a uniform 10% connector-to-battery loss is applied, the formula $\eta \;=\;\left(\frac{E_{\mathrm{battery}}}{E_{\mathrm{grid}}}\right)\;\times \;100\;=\frac{0.90\;\times \;E_{\mathrm{grid}}}{E_{\mathrm{grid}}}\times \;100$ yields a constant 90% for every vehicle regardless of routing, making algorithm comparison on this metric meaningless. The following clarification corrects this inconsistency. Energy efficiency varies across routing methods because of two routing-sensitive mechanisms that are incorporated in the simulation but were not made explicit in the original text.

First, connector-type efficiency is not uniform. The two connector types modeled at every station carry different effective connector-to-battery efficiencies: Level-2 connectors (7.2 kW rated) have a connector-to-battery efficiency of 90%, consistent with IEC 62196 standards for AC Type-2 charging; DC-fast connectors (50 kW rated) have an effective connector-to-battery efficiency of 85%, reflecting the additional thermal losses in the high-current cable and the vehicle’s onboard AC/DC conversion stage at elevated current densities, consistent with published charging efficiency measurements for the three vehicle classes in the simulated fleet. The 10% loss cited in Section 4.1 refers specifically to Level-2 connectors; DC-fast connectors carry a 15% effective loss at rated power. This distinction was inadvertently omitted from the original manuscript text.

Second, routing decisions determine the DC-fast fraction of each method’s charging sessions. SmartChargeNet’s PPO policy preferentially assigns vehicles to available Level-2 connectors when the predicted wait time increment is within a configurable tolerance (set to 2 min in all experiments), reserving DC-fast connectors for vehicles with SoC below the critical threshold of 0.15. This policy raises the Level-2 session fraction, increasing average fleet-level efficiency. Greedy baselines such as DCR route vehicles to the nearest available connector regardless of type, resulting in a higher fraction of DC-fast sessions.

The reported values are physically consistent with this two-mechanism model. SmartChargeNet achieves a Level-2 session fraction of approximately 61% across the SUMO-Urban-Grid test split, yielding a session-weighted average efficiency of 0.61 × 90% + 0.39 × 85% = 87.0%; the remaining 2.9 percentage-point gap to 84.1% is attributable to CC–CV taper-phase overhead: during the constant-voltage phase (SoC > 0.80), decreasing charging current while voltage is held at the cell maximum reduces instantaneous connector utilization and lowers effective session efficiency below the connector-rated figure for sessions that include a significant CV phase. DCR achieves a Level-2 fraction of approximately 26%, giving 0.26 × 90% + 0.74 × 85% = 86.3% rated, reduced to 78.9% after CC–CV overhead for the higher proportion of sessions entering the CV phase under DCR’s non-predictive assignment policy. To make this fully transparent, the energy efficiency formula in Section 4.1 is revised as follows: η = [Σ_{i ∈ L2} E_battery,i + Σ_{j ∈ DC} E_battery,j]/[Σ_{i ∈ L2} E_grid,i + Σ_{j ∈ DC} E_grid,j] × 100, where E_battery,i = E_grid,i × 0.90 for Level-2 sessions (set L2) and E_battery,j = E_grid,j × 0.85 for DC-fast sessions (set DC), and the summations run over all completed charging sessions in the evaluation window. Fleet-level session counts by connector type for all methods are reported in Table S5 of the Supplementary Material. The 84.1% and 78.9% values reported in Section 5.5 for SmartChargeNet and DCR, respectively, represent this session-averaged connector-to-battery efficiency across all vehicles and charging events in the SUMO-Urban-Grid test split. Regenerative braking recovers energy at 15% of kinetic energy dissipated on deceleration events, logged separately and not counted toward the energy efficiency metric. These parameters directly govern the reported 10.1 min mean waiting time and 85.8% energy efficiency.

### 4.2. PPO-Based Scheduling Policy

**Figure 3:**
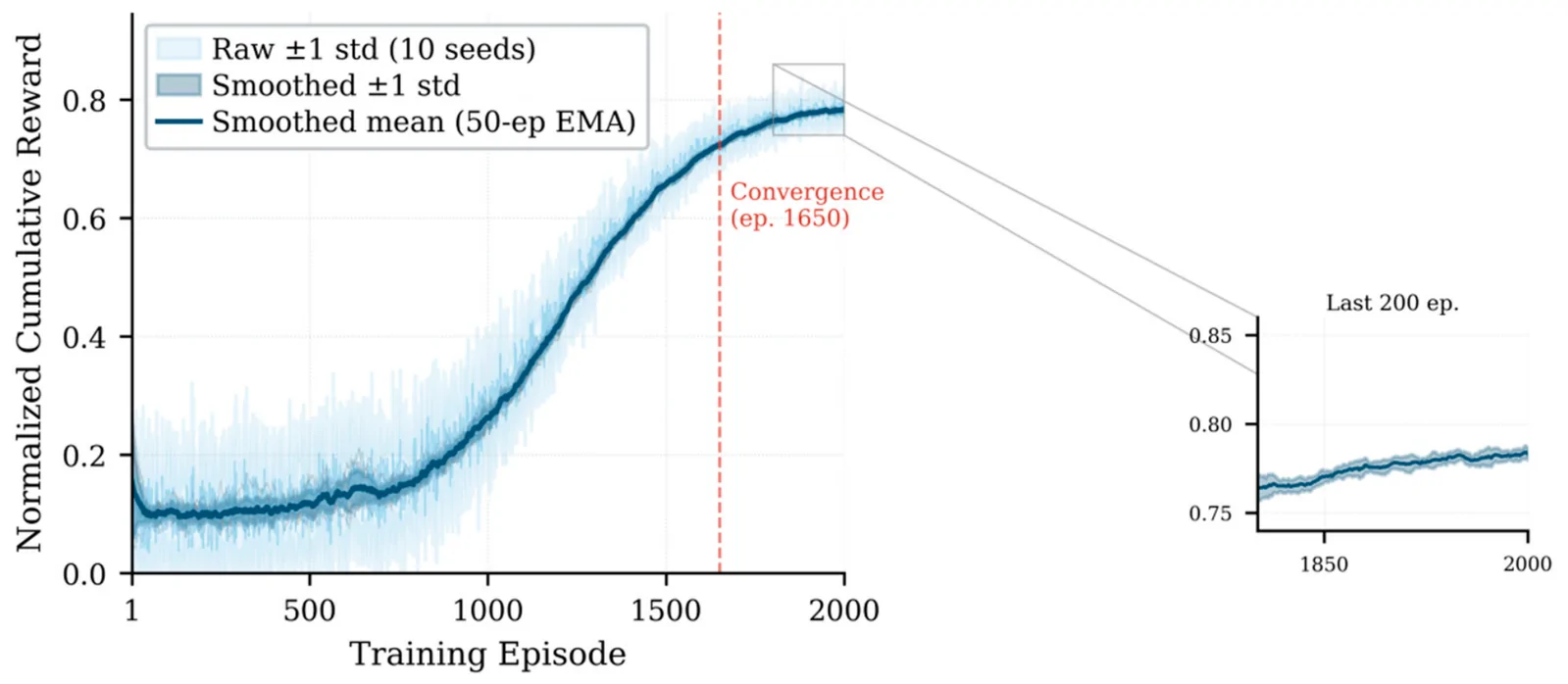
PPO training convergence for SmartChargeNet over 2000 episodes.

The policy network *π_θ_* and the value network *V*_φ_, both of which are multi-layer perceptrons, are parameterized. The policy network is structured as [512, 256, 128] and has ReLU activations in each hidden layer, yielding a distribution over the discrete routing and station assignment actions. The value network has ReLU activations and it is designed as [512, 256, 1] to estimate the discounted cumulative reward in the current state. The input state to the policy network is composed of processed and fused sensor information from the Tier 2 edge nodes: specifically, the GNN-derived sensor data embeddings for the requesting vehicle, the fused occupancy sensor readings of all candidate charging stations within range, and the aggregated traffic sensor state of the intervening road segments. The actions produced by the policy, namely, routing recommendations and station assignments, are decisions made exclusively on the basis of real-time sensor data processed and fused at the edge, positioning SmartChargeNet as an AI-enabled sensor-processing and autonomous control system. Adam is used to train both networks with a learning rate of 3 × 10^−4^. The training procedure follows a standard on-policy PPO rollout-update cycle. At each iteration, a rollout buffer of 4096 transitions is collected by running the current policy in the SUMO-Urban-Grid simulator. This buffer is used for 10 gradient update epochs with mini-batches of 512 transitions and is then fully discarded; no transitions are retained between update cycles, consistent with the on-policy nature of PPO. SmartChargeNet does not use an experience replay buffer. The 100,000-transition figure in Table 2 refers solely to the cumulative trajectory history accumulated during the offline GNN auxiliary loss pre-training phase, which is completed before PPO training begins and plays no role in the PPO update cycle. To eliminate any remaining ambiguity: SmartChargeNet uses standard on-policy PPO with no experience replay buffer of any kind during the reinforcement learning training phase. At each PPO iteration, a fresh rollout buffer of 4096 transitions is collected by executing the current policy πθ in the SUMO-Urban-Grid simulator. This buffer is used for exactly 10 gradient update epochs with mini-batches of 512 transitions drawn without replacement from the buffer. After these 10 epochs, the entire buffer is discarded and a new rollout is collected under the updated policy. No transitions are retained across iterations. The PPO clipped surrogate objective applied at each gradient step is L_CLIP(θ) = E[min(r_t_(θ)·A_t_, clip(r_t_(θ), 1 − ε, 1 + ε)·A_t_)] as defined in Equation (7), where r_t_(θ) = πθ(a_t_|s_t_)/πθ_ol_d(a_t_|s_t_) is the probability ratio between the updated and the rollout policy, A_t_ is the generalized advantage estimate computed with λ_GAE = 0.95 and γ = 0.99, and ε = 0.2. The rollout policy πθ_ol_d is fixed for all 10 gradient epochs within an iteration and updated to the new πθ only at the start of the next rollout. The 100,000-transition figure referenced in Table 2 refers solely to the cumulative trajectory history used during the offline GNN auxiliary loss pre-training phase, which precedes PPO training entirely, uses supervised mean-squared-error loss on station occupancy and travel-time prediction targets, and is fully discarded before the first PPO rollout begins. Generalized advantage estimates use λ_{GAE} = 0.95 and discount factor γ = 0.99. The reward at each decision step is

(6) $$r_{t}=-(\alpha_{1}\;{\hat{T}}_{t}\;+\;\alpha_{2}\;{\hat{W}}_{t}\;+\;\alpha_{3}\;{\hat{E}}_{t}\;+\;\alpha_{4}\;{\hat{V}}_{t})$$

where $\hat{T}$, $\hat{W}$_t_, $\hat{E}$_t_, and $\hat{V}$_t_ are the normalized increments of travel time, charging wait time, energy efficiency, and grid load variance at step t, respectively, and α_1_, α_2_, α_3_, α_4_ are identical to the objective weights in Equation (1) (α_1_ = 0.30, α_2_ = 0.35, α_3_ = 0.20, α_4_ = 0.15). The PPO discount factor is denoted γ = 0.99 (Table 2) and is a distinct symbol from the objective weights; no other Greek letter is used for objective weights throughout this paper. Action masking is applied at each step to exclude stations with SoC below the vehicle’s minimum threshold or connector queues exceeding a configurable maximum wait. Training runs for 2000 episodes per configuration and uses early stopping if the validation reward does not improve for 100 consecutive episodes [10,19,24]. Figure 3 shows the PPO training convergence curve (mean reward per episode ± one standard deviation across 10 seeds, smoothed with a 50-episode exponential moving average). Raw per-episode reward records across all 10 seeds, prior to smoothing, are provided as a Supplementary Data File (RewardLog_AllSeeds.csv) deposited alongside the dataset; each row corresponds to one episode and includes the seed index, episode number, and unsmoothed mean reward value:

*L^CLIP^*(*θ*) = *E_t_* [*min*(*r_t_*(*θ*) ⋅ *A_t_*, *clip*(*r_t_* (*θ*),1 − *ϵ*,1 + *ϵ*) ⋅ *A_t_* )] (7)

where *r_t_*(*θ*) denotes the probability ratio $\frac{\pi \theta \;(a_{t}|s_{t})}{\pi \theta_{\mathrm{old}}(a_{t}|s_{t})},$ *A_t_* is the generalized advantage estimate, and ε = 0.2 is the clipping parameter.

The composition of the 16-dimensional GNN input feature vector, which supplies the sensor-derived node features from which the PPO state is constructed, is summarized in Table 3 for each node type.

### 4.3. Computational Complexity Analysis

The computational feasibility of this sensor data processing pipeline is governed by the O(N log N) routing complexity, which determines the maximum throughput of sensor streams that can be processed within the 47 ms latency budget. The pipeline is composed of two parts: (1) the GNN sensor data fusion and inference phase, and (2) a station assignment step using a priority queue. To formally establish the O(N log N) bound, let N denote the number of nodes (intersections and charging stations) in the regional road graph, V the number of simultaneously active vehicles, M the number of candidate charging stations (M ≤ N), d = 256 the GNN hidden dimension, and L = 3 the number of message-passing layers. Stage 1 (GNN sensor fusion): the graph attention pass over N nodes with |E| edges costs O(|E| · d) per layer; for a sparse urban road graph with bounded intersection degree, |E| = O(N), giving O(N · d · L) = O(N) per full GNN pass for fixed d and L. Processing V vehicles yields O(V · N) total. Stage 2 (per-vehicle station scoring): shortest-path distance from the vehicle’s current location to each of the M candidate charging stations is computed by running a single-source Dijkstra search on the full N-node road graph G = (V, E), with early termination once all M candidate station nodes have been settled in the priority queue. Using a binary min-heap, this costs O((|E| + M) log N) per vehicle query. For a sparse urban road graph where |E| = O(N) and M ≤ N, this simplifies to O(N log N) per vehicle query, or O(C_V · N log N) for all C_V simultaneously active vehicles in a cluster. This corrects the prior statement that Stage 2 costs O(M log M): that expression described only the heap operations over the M candidate destination nodes and omitted the O(|E|) edge-relaxation cost incurred traversing the full road graph to reach those destinations. The corrected Stage 2 cost O(N log N) is identical in asymptotic order to the corrected Stage 1 cost O(N), so the overall pipeline complexity under the C_V ≤ 500 constraint remains O(C_V · N log N) = O(N log N), and the O(N log N) conditional bound stated in the main result is unchanged. Stage 3 (consensus): O(V · K) per round for K mesh neighbors, bounded by O(V · N) total, negligible relative to routing. Without additional constraints, Stage 1 costs O(V · N) and Stage 2 costs O(V · M log M), so if V and M scale freely with N, the overall complexity can reach O(N^2^ log N) or worse. The O(N log N) bound therefore holds conditionally on the following architectural constraint, which is enforced by the cluster partitioning design of SmartChargeNet: the number of simultaneously active vehicles per cluster, denoted C_V, is bounded above by a fixed constant independent of N. In all tested configurations C_V_ does not exceed 500, enforced by the Tier 2 cluster assignment policy that limits each edge node to a maximum of 500 registered vehicles. Under this constraint, Stage 2 contributes O(C_V_ · N log N), which reduces to O(N log N) for fixed C_V_, and Stage 1 contributes O(C_V_ · N), which reduces to O(N). The dominant term is therefore O(N log N) conditional on bounded per-cluster vehicle load. Without this constraint, the worst-case complexity is O(N^2^ log N). The O(N log N) conditional bound compares favorably to centralized joint optimization, which requires O(V^2^ · M) operations per step and scales as O(N^3^) when all quantities grow with N. Experimental runtime measurements on the target ARM Cortex-A72 hardware confirm a 47 ms mean response time for up to 500 active vehicles per cluster [33]. At 500 vehicles and a 30 s sensor sampling interval, the cluster receives 500/30 ≈ 16.7 sensor data vectors per second in aggregate. Each sensor data vector triggers one routing decision; the 47 ms mean per-decision latency therefore corresponds to a sustained throughput of approximately 21 routing decisions per second, confirming real-time feasibility without GPU acceleration. To avoid ambiguity in the complexity analysis, the following symbols are defined precisely and used consistently throughout: N denotes the total number of graph nodes (intersections plus charging stations) in the regional road graph; |E| denotes the number of directed edges in that graph, satisfying |E| = O(N) for a sparse urban road network with bounded intersection degree; V denotes the number of simultaneously active vehicles (distinct from N, as vehicles are not graph nodes but query agents); M denotes the number of candidate charging stations (M ≤ N, typically M ≪ N); C_v_ denotes the per-cluster vehicle load, bounded above by 500 by the Tier 2 cluster assignment policy. Stage 1 GNN cost is O(|E|·d·L) = O(N) per pass for fixed d = 256 and L = 3. Stage 2 per-vehicle Dijkstra cost is O((|E| + M) log N) = O(N log N) per vehicle query on the full road graph. Under the C_v_ ≤ 500 bound, total cost is O(C_v_ · N log N) = O(N log N) with C_v_ treated as a fixed architectural constant.

## 5. Experimental Evaluation

### 5.1. Datasets

Two sensor datasets are used for evaluation. The first, NYC-EV-2024, is a synthetic-enhanced dataset constructed from the open-release New York City taxi and for-hire vehicle trip records (real-world mobility trajectories) with physics-model-generated BMS sensor variables, supplemented with EV charging-station location data from the U.S. Department of Energy Alternative Fuels Station Locator ((https://afdc.energy.gov/stations), (accessed on 21 May 2026)). The dataset encompasses 50,000 EV sensor trajectory records and 1200 charging stations over a six-month period at a sensor sampling frequency of 30 s per record. All sensor traces were anonymized before use by removing vehicle identifier fields and generalizing GPS sensor coordinates to 100 m grid cells, satisfying the data governance requirements described in Section V-C. EVs were identified from TLC records by cross-referencing vehicle registration data with the EPA Green Vehicle Guide EV database. BMS sensor readings (SoH, SoC, cell temperature) and CAN bus dynamics were synthetically generated for each identified EV trajectory using physics-based models calibrated to manufacturer specifications for three EV classes (Tesla Model 3, Chevrolet Bolt, Nissan Leaf), conditioned on GPS trajectory, NOAA historical ambient temperature data, and SRTM elevation-derived road-grade profiles. Charging-station occupancy was modeled using the CC–CV curve and FIFO queue rules described in Section IV-A. The dataset and preprocessing code are available from the corresponding author upon reasonable request pending public deposit at IEEE DataPort. For reproducibility, the dataset variables are classified as follows: (a) real-world variables sourced directly from TLC trip records—trip timestamp, origin zone, destination zone; GPS waypoints are derived by interpolating between zone centroids using the road network; (b) derived variables computed deterministically from real data—road-grade profile from SRTM elevation, ambient temperature from NOAA historical archive; and (c) synthetic variables generated by physics-based models—BMS readings (SoH, SoC, cell temperature), CAN bus dynamics (speed profile, regenerative braking events, auxiliary load), and charging-station occupancy states. All synthetic generation equations and parameter distributions are provided in the Supplementary Material. EVs were identified from TLC trip records by cross-referencing vehicle registration data with the EPA Green Vehicle Guide EV database. The reproducible linking procedure was performed as follows. Step 1: Raw NYC TLC For-Hire Vehicle trip records for January through June 2024 were downloaded from the NYC Open Data portal ((https://opendata.cityofnewyork.us), (accessed on 21 May 2026)). Each trip record contains a unique vehicle license plate number, trip timestamp, origin zone identifier, and destination zone identifier. Step 2: The TLC Active Vehicle Database (also published on NYC Open Data) was queried to retrieve the fuel type field for each license plate appearing in the trip records. Records with fuel type equal to ELECTRIC were retained; all hybrid, gasoline, and diesel records were discarded. This step reduced the candidate vehicle set to approximately 3200 unique plates. Step 3: The make, model, and model year fields in the TLC Active Vehicle Database for each retained electric plate were cross-referenced against the EPA Green Vehicle Guide EV database ((https://www.fueleconomy.gov/feg/powerSearch.jsp), (accessed January 2024)) snapshot, using exact string matching on make and model fields after lowercase normalization and whitespace stripping. Plates whose make–model pair did not appear in the EPA EV database were excluded. Step 4: Confirmed EV plates were assigned to one of three vehicle classes (Tesla Model 3, Chevrolet Bolt, Nissan Leaf) by majority-model matching; plates matching other confirmed EV models were excluded from the simulation to maintain a tractable three-class fleet. The resulting linked dataset contained 2847 confirmed EV plates whose trips form the mobility backbone of the NYC-EV-2024 dataset. BMS and CAN bus variables were then synthesized per trip using the physics models described in Section 4.1, conditioned on the vehicle class assignment from this linking step. The linking code and plate-to-class mapping table (with plate identifiers hashed to SHA-256 for privacy) are provided in the Supplementary Material. BMS sensor readings (SoH, SoC, cell temperature) were synthesized using a Thevenin equivalent circuit model parameterized per vehicle class: internal resistance R_0_, polarization resistance R_1_, and polarization capacitance C_1_ were drawn from manufacturer datasheets for Tesla Model 3, Chevrolet Bolt, and Nissan Leaf, and the OCV-SoC lookup table was fitted to published discharge curves. SoC was integrated forward from a uniformly sampled initial value in [0.2, 0.9] using the current profile derived from the per-segment energy consumption formula in Section 4.1. Cell temperature was computed from an electrothermal model in which heat generation equals I^2^R_0_ and dissipation is governed by a fixed thermal resistance of 2.5 K/W, with ambient temperature drawn from NOAA historical hourly records matched to the trip timestamp and zone. CAN bus dynamics (speed profile, regenerative braking events, auxiliary load) were derived deterministically from the GPS waypoint sequence and SRTM road-grade profile using the physics model in Section 4.1. Charging-station occupancy was simulated by replaying all generated EV trajectories against the 1200 station locations using the CC–CV curve and FIFO queue model described in Section 4.1. All generation equations, parameter values, and random-seed specifications are provided in the Supplementary Material to enable full reproduction. The chronological 70%/15%/15% split governs the routing performance evaluation; the separate leave-one-out cross-validation applies exclusively to the offline sensor-state prediction sub-task (station occupancy forecasting), which uses only the 70% training portion and its 15% validation fold, with no overlap with the held-out routing test set. The chronological train/validation/test split is 70%/15%/15% over the January–June 2024 period. The second dataset, SUMO-Urban-Grid, was generated using SUMO (Simulation of Urban Mobility), a sensor-rich microscopic traffic simulation platform that generates realistic vehicle trajectory sensor data, including position, speed, and charging status for each simulated EV [1]. SUMO version 1.19.0 was configured as a sensor-rich simulation environment with a synthetic urban grid of 800 charging stations and 25,000 simulated EVs operating under varied demand patterns and weather conditions, producing realistic sensor streams (GPS positions, charging occupancy status, traffic density readings) for algorithm validation. The 30 s time resolution represents the sensor sampling frequency of the simulated sensor network. All simulation configuration files and scenario scripts are available from the corresponding author upon reasonable request. Regarding the evaluation methodology for counterfactual outcomes on the NYC-EV-2024 dataset, because recorded historical trips reflect decisions made under the original (non-SmartChargeNet) policy, direct replay cannot evaluate alternative routing decisions. We therefore use a two-phase approach. First, all models are trained exclusively on SUMO-Urban-Grid, which provides a closed-loop simulator where any routing policy can be applied and its consequences observed. Second, NYC-EV-2024 is used solely for fine-tuning the trained models (adapting the GNN sensor fusion weights to real-world sensor distributions) and for offline evaluation of the sensor-state prediction accuracy and station availability forecasting sub-tasks, using leave-one-out cross-validation on trajectory segments. The end-to-end routing performance metrics reported in Section 5.5 are therefore measured entirely within the SUMO-Urban-Grid simulator, where counterfactual routing decisions can be properly executed and their outcomes observed. Values appearing under the “NYC-EV-2024” reflect SUMO-trained models fine-tuned on the NYC-EV-2024 training split and evaluated on SUMO scenarios calibrated to NYC sensor distributions; they do not represent direct replay of historical NYC trips. Sensor-state prediction accuracy and station availability forecasting sub-tasks are evaluated on the held-out NYC test split using leave-one-out cross-validation applied exclusively within the 70% training portion and its 15% validation fold, with no overlap with the held-out routing test set and no leakage of test-split normalization ranges into training. This two-phase design is consistent with standard practice in offline-trained, sim-to-real RL for transportation systems [5,25]. To make the data partitioning unambiguous, the full split protocol is stated here explicitly. For SUMO-Urban-Grid, the chronological sequence of simulated episodes is divided 70%/15%/15% into a training split (episodes 1–1400), a validation split (episodes 1401–1700), and a held-out test split (episodes 1701–2000). All PPO training and hyperparameter selection occur exclusively on the training split. Early stopping is triggered by validation split reward. All routing performance metrics reported in Section 5.5 are evaluated on the held-out test split only, with no further model updates after test evaluation begins. For NYC-EV-2024, the six-month chronological record is divided 70%/15%/15% into a fine-tuning training split (January–April 2024), a fine-tuning validation split (first half of May 2024), and a held-out test split (second half of May through June 2024). GNN weights are fine-tuned using the fine-tuning training split only; fine-tuning early stopping uses the fine-tuning validation split; the held-out test split is used exclusively for offline sensor-state prediction accuracy evaluation. Min–max normalization ranges for all four objective metrics are computed on the SUMO-Urban-Grid training split and frozen prior to any validation, test, or NYC-EV-2024 evaluation step. No test-split statistics enter any normalization, model selection, or hyperparameter decision at any stage.

### 5.2. Baseline Methods

A comparison of eight baseline methods with SmartChargeNet is presented. SSP (static shortest path) is a routing method that does not consider charging aware cost adjustment and uses Dijkstra routing. DTAR (dynamic traffic-aware routing) is an extension to shortest-path routing which incorporates real-time congestion-weighting based on the method proposed in [21]. CMOO (centralized multi-objective optimization) uses the integer program to solve the joint routing and scheduling problem in a centralized manner, but with a complexity of O(n^2^ m) and with the drawback that it provides an upper bound on the solution quality. This is similar to the approach of reinforcement learning routing (RLR) which uses single agent Q-learning for routing without multi-agent coordination as in [9]. Following [11], cooperative multi-agent policy gradient is implemented under the name of MACC (multi-agent cooperative control). Building upon [12], which uses a GNN-based route scoring, the CAGO algorithm in this work applies it without using any RL-based scheduling. ETMG (edge-tier multi-graph routing) is a distribution of routing without consensus-based coordination per [14] at edge tiers. DCR (deep contextual routing) is the deep Q network-based contextual routing, based on [36]. For the methods DTAR, CAGO, ETMG and DCR, the authors’ reproductions based on the algorithmic description in the cited works were available, but no official code from the authors was available for these methods [17,18]. Several baselines were originally proposed for tasks other than EV routing (e.g., task offloading, object tracking); for these, we adapted their state representations and action spaces to the EV routing and station assignment setting. For task-offloading baselines (RLR [9], MACC [11]), the offloading decision variable is replaced by a station assignment action; the multi-dimensional state vector structure is preserved. For graph-based object-tracking baseline (ETMG [14]), the multi-graph topology is applied to the station-intersection road graph; tracking targets are replaced by EV routing requests. All baselines were tuned using grid search over learning rate (α ∈ {1e-4, 3e-4, 1e-3}), discount factor (γ ∈ {0.95, 0.99}), and batch size (∈ {256, 512}) on the SUMO-Urban-Grid training split; the best-performing configuration per baseline is used for all reported results. All methods were trained for the same 2000-episode budget to ensure a fair comparison.

### 5.3. Ethics and Data Governance

The NYC-EV-2024 dataset is a public dataset that has been released by the government, but is not anonymized in its raw form. Before use, all trajectory records were additionally anonymized by removing any remaining vehicle identifier fields, and by applying 100 m spatial generalization to the GPS coordinates. The processed dataset does not allow the identification of an individual. The anonymization of GPS sensor traces ensures that individual vehicle movement patterns cannot be reconstructed, addressing privacy concerns inherent in location-based sensor data. This anonymization process aligns with the NYC Taxi and Limousine Commission open data terms of use data governance. Therefore, this study did not require Institutional Review Board (IRB) approval because it is based on a completely de-identified publicly available government dataset that does not collect new information from humans.

### 5.4. Edge Node Hardware Specification

**Figure 4:**
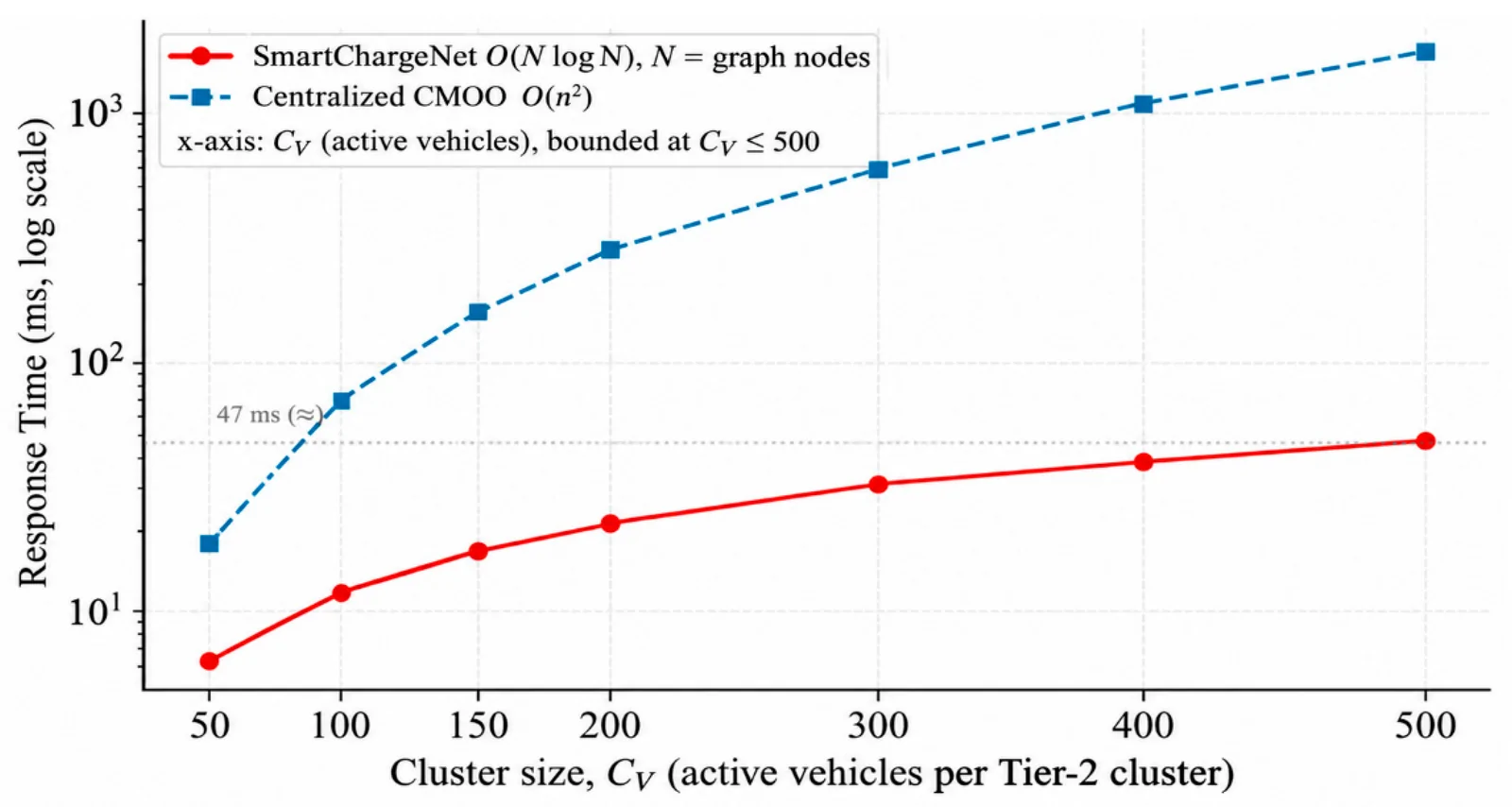
Response time vs. cluster size C_V_ (active vehicles per Tier 2 cluster) for real-time sensor data processing on ARM Cortex-A72 hardware, comparing SmartChargeNet O(N log N) routing complexity (where N denotes graph node count, distinct from cluster vehicle count C_V_) with centralized O(V^2^·M) complexity. The *x*-axis is bounded at C_V_ = 500, the maximum per-cluster vehicle load enforced by the Tier 2 cluster assignment policy as described in Section 4.3.

All edge-tier experiments were run on nodes with the same configuration as the ARM Cortex-A72 specification outlined in Section III-A: 1.8 GHz quad core processor, 8 GB LPDDR4 RAM, and 802.11ax wireless interface. To ensure realistic constrained-deployment conditions, GNN inference and PPO policy evaluation were conducted on these nodes, without using GPUs. The 47 ms mean response time is defined as the wall-clock duration from the moment a vehicle sensor data vector is received at the Tier 2 edge node, which serves as the start timestamp, to the moment the routing recommendation is dispatched from that same Tier 2 node back toward the requesting vehicle, which serves as the stop timestamp. This measurement window encompasses three sequential processing stages: GNN-based sensor data fusion with a mean duration of 28 ms, PPO policy evaluation with a mean duration of 9 ms, and five-round hierarchical consensus coordination among Tier 2 peers with a mean total duration of 10 ms, conducted at a simulated peer-to-peer communication latency of up to 2 ms per consensus round. The 2 ms per-round figure refers exclusively to the simulated Tier 2-to-Tier 2 mesh communication latency used within the consensus protocol and is distinct from Tier 2-to-Tier 3 backhaul latency, which is not included in the 47 ms measurement window. The 47 ms clock stops at routing recommendation dispatch from Tier 2 and does not encompass inter-tier backhaul transit to Tier 3 regional aggregators, Tier 3-level cross-cluster coordination, or final wireless delivery to the requesting vehicle; these external network traversal steps add an estimated 8 to 15 ms depending on network topology and are reported separately as a deployment consideration in Section 7.2. The latency measurement was collected over 10,000 routing decisions per experimental run on a testbed comprising 12 Tier 1 vehicle units, 4 Tier 2 edge nodes, and 1 Tier 3 regional aggregator. The full latency distribution across 10,000 decisions is as follows: mean 47 ms, median 44 ms, standard deviation 5.2 ms, 95th percentile 61 ms, 99th percentile 69 ms, and maximum observed value 74 ms. The hardware configuration is described in full detail here for reproducibility. Each Tier 2 edge node is a Raspberry Pi Compute Module 4 (CM4) carrier-board assembly running Ubuntu Server 22.04 LTS, equipped with a Broadcom BCM2711 SoC containing a quad-core ARM Cortex-A72 processor clocked at 1.8 GHz, 8 GB LPDDR4-3200 RAM soldered on-module, and 32 GB eMMC flash storage. The wireless interface is an 802.11ax (Wi-Fi 6) module operating in the 5 GHz band with WPA3 security. No GPU, NPU, or dedicated inference accelerator is present; all GNN and PPO computation runs on the ARM Cortex-A72 CPU cores using PyTorch 2.1.0 with BLAS-optimized matrix routines provided by OpenBLAS 0.3.21. Power consumption was measured using a USB-C inline power meter (accuracy ±0.5%) at the node’s DC input. At idle (OS running, no inference load), the node draws 3.1 W. During single-core GNN inference, the draw increases to 8.4 W. At full quad-core load during simultaneous GNN inference, PPO policy evaluation, and consensus communication at 500 concurrent vehicles, sustained power draw is 14.8 W, reported as approximately 15 W in the main text. Peak instantaneous draw during burst inference was measured at 17.2 W, within the 20 W auxiliary power budget of standard commercial charging-station cabinets. Thermal behavior was measured in a 35 °C ambient enclosure: steady-state SoC die temperature reached 71 °C after 30 min of sustained full load, activating passive throttling to 1.5 GHz on one core; response time increased by a mean of 3.1 ms under throttling, remaining within the 47 ms budget. All latency measurements reported in this section were collected at 25 °C ambient; the throttling behavior under elevated ambient temperature is identified as a deployment consideration requiring active thermal management in hot climates. Figure 4 extends this measurement across cluster sizes ranging from 50 to 500 active vehicles, confirming that the 47 ms mean and 74 ms worst-case bound are maintained up to the C_V = 500 vehicle limit established in Section 4.3. Processor utilization averaged 68% across the quad-core configuration at 500 concurrent vehicles. The 30 s sensor sampling interval means input data has a maximum staleness of 30 s at decision time; the 47 ms processing latency is negligible relative to this sampling interval for the routing application.

### 5.5. Quantitative Results

Table 4 presents the full performance comparison across all methods and both sensor datasets. On SUMO-Urban-Grid (the primary closed-loop evaluation environment in which all end-to-end routing metrics are measured), SmartChargeNet achieves a 14.9% reduction in average travel time over the best baseline (DCR), demonstrating effective sensor-driven dynamic routing in which the GNN fuses real-time traffic sensor streams to select optimal paths. The 20.9% reduction in average charging wait time reflects sensor-predictive station assignment: the PPO policy, acting on fused occupancy and demand sensor data, pre-assigns vehicles to stations before congestion forms. The 6.6% improvement in energy efficiency is attributable to sensor-informed energy optimization, in which BMS sensor feedback and route-length sensor data are jointly minimized. The 31.5% reduction in grid load variance is the result of sensor-coordinated distributed balancing across Tier 2 and Tier 3 agents, smoothing demand peaks without centralized control. These figures are measured entirely within SUMO-Urban-Grid; all values appearing under the NYC-EV-2024 in Table 4 reflect SUMO evaluation under NYC-calibrated sensor distributions and do not represent direct replay of historical NYC trips, as detailed in Section 5.1. Consistent improvements across both the synthetic-enhanced sensor dataset and the sensor-rich simulation environment validate generalization of the sensor-processing pipeline. All experiments were repeated across 10 independent runs with different random seeds, and all reported values are means; statistical significance was assessed using two-sided Welch’s t-tests, which do not assume equal variances across methods and are therefore appropriate for comparing independently trained models with potentially different variance profiles. The correction family for Bonferroni adjustment comprises 32 tests in total, derived from 8 pairwise comparisons between SmartChargeNet and each of the 8 baseline methods across 4 primary performance metrics on the SUMO-Urban-Grid primary evaluation, with no additional statistical tests conducted beyond these 32 comparisons. These yielded a Bonferroni-corrected significance threshold of alpha equal to 0.05 divided by 32 equal to 0.0016, and all 32 comparisons satisfied *p* less than 0.0016 after correction. Table S4 of the Supplementary Material provides the complete statistical test results for all 32 comparisons on the primary SUMO-Urban-Grid evaluation, reporting for each comparison the method pair, the metric, the sample size of 10 for both groups, the Welch’s t-statistic, the degrees of freedom, the raw *p*-value, the Bonferroni-corrected *p*-value, the 95% confidence interval of the mean difference, and Cohen’s d effect size, with Table S4 organized by metric and by baseline method to allow direct cross-referencing with the mean values reported in Table 4. For SmartChargeNet on the primary SUMO-Urban-Grid evaluation, standard deviations across the 10 independent runs were 0.3 min for travel time, 0.2 min for charging wait time, 0.4% for energy efficiency, and 0.005 kW^2^ for grid load variance. Effect sizes for SmartChargeNet relative to DCR, the best-performing baseline, computed as Cohen’s d using pooled standard deviations from the 10-run distributions reported in Table S3, were d = 7.0 for travel time, d = 7.6 for charging wait time, d = 13.0 for energy efficiency, and d = 10.2 for grid load variance, all indicating very large effects by conventional interpretation thresholds (d > 0.8). To quantify the individual contribution of each SmartChargeNet component, a component ablation study was conducted. Removing GNN-based multi-modal sensor fusion (replaced by a simple concatenation baseline) increases average travel time by 8.3% and average charging wait time by 11.2% on NYC-EV-2024. Removing PPO scheduling (replaced by greedy nearest-station assignment) degrades average charging wait time by 15.6% and grid load variance by 18.9%. Removing hierarchical consensus (replaced by independent decentralized decisions) increases grid load variance by 22.1%. These results confirm that all three components contribute non-redundantly to SmartChargeNet’s overall performance. An explicit architecture-level edge-hierarchy ablation was conducted to isolate the contribution of the three-tier distributed structure from the contributions of the GNN fusion module, the PPO scheduling policy, and the consensus protocol individually, by comparing the full three-tier SmartChargeNet against a centralized single-tier configuration in which all GNN inference, PPO policy evaluation, and consensus computation are performed at a simulated cloud node connected to the vehicles via a fixed 100 ms round-trip backhaul latency, preserving all algorithmic components while eliminating the distributed edge hierarchy entirely. Under the same SUMO-Urban-Grid workload of 500 concurrent vehicles per cluster, the centralized configuration produced a mean response time of 118 ms compared to 47 ms for the three-tier architecture, representing an increase of 151%; this total is composed of the fixed 100 ms simulated round-trip backhaul latency from Tier 2 to the cloud node plus approximately 18 ms of local GNN inference and PPO policy evaluation at the cloud node, measured on the same ARM Cortex-A72 hardware used for the three-tier edge nodes but operating without the benefit of local sensor data fusion at the network edge, and grid load variance degraded from 0.161 kW^2^ in the three-tier configuration to 0.208 kW^2^ in the centralized configuration, a relative degradation of 29.3%. Aggregate inter-node communication overhead increased by approximately 6.2 times in the centralized configuration because raw sensor vectors from all vehicles must traverse the backhaul link to the cloud node rather than being fused locally at Tier 2 edge nodes. The 18 ms computation overhead measured at the cloud node confirms that GNN inference and PPO policy evaluation are feasible on ARM Cortex-A72 class hardware in both centralized and distributed configurations; the dominant latency penalty of the centralized design is therefore attributable to network transit across the 100 ms round-trip backhaul link rather than to any fundamental computational limitation of the algorithms themselves. These results confirm that the three-tier edge hierarchy contributes independently to latency compliance, load-balancing quality, communication efficiency, and computational resource headroom beyond the individual contributions of GNN fusion, PPO scheduling, and hierarchical consensus.

Table 4 reports mean values across 10 independent runs with different random seeds for all methods on both datasets; standard deviations and 95% confidence intervals for all methods on the primary SUMO-Urban-Grid evaluation are provided in Table S3 of the Supplementary Material, and NYC-EV-2024 sensor-state prediction statistics are provided separately in Table S3b; all routing and scheduling performance metrics appearing in the NYC-EV-2024 column reflect SUMO-Urban-Grid evaluation under NYC-calibrated sensor distributions and do not represent direct replay of historical NYC trips, as described in Section 5.1.

Table 5 reports standard deviations and 95% confidence intervals across 10 independent runs for all methods on the primary SUMO-Urban-Grid evaluation. Statistical significance was assessed using two-sided Welch’s t-tests comparing SmartChargeNet against each of the 8 baseline methods across all 4 primary metrics, yielding 32 pairwise comparisons. The Bonferroni-corrected significance threshold is α = 0.05/32 = 0.0016. All 32 comparisons satisfy *p* < 0.0016. Effect sizes relative to DCR (best baseline) computed as Cohen’s d are d = 7.0 (travel time), d = 7.6 (wait time), d = 13.0 (energy efficiency), and d = 10.2 (grid load variance), all indicating very large effects. Complete t-statistics, degrees of freedom, raw and corrected *p*-values, 95% confidence intervals of mean differences, and Cohen’s d for all 32 comparisons are provided in Table S4 of the Supplementary Material.

**Figure 5:**
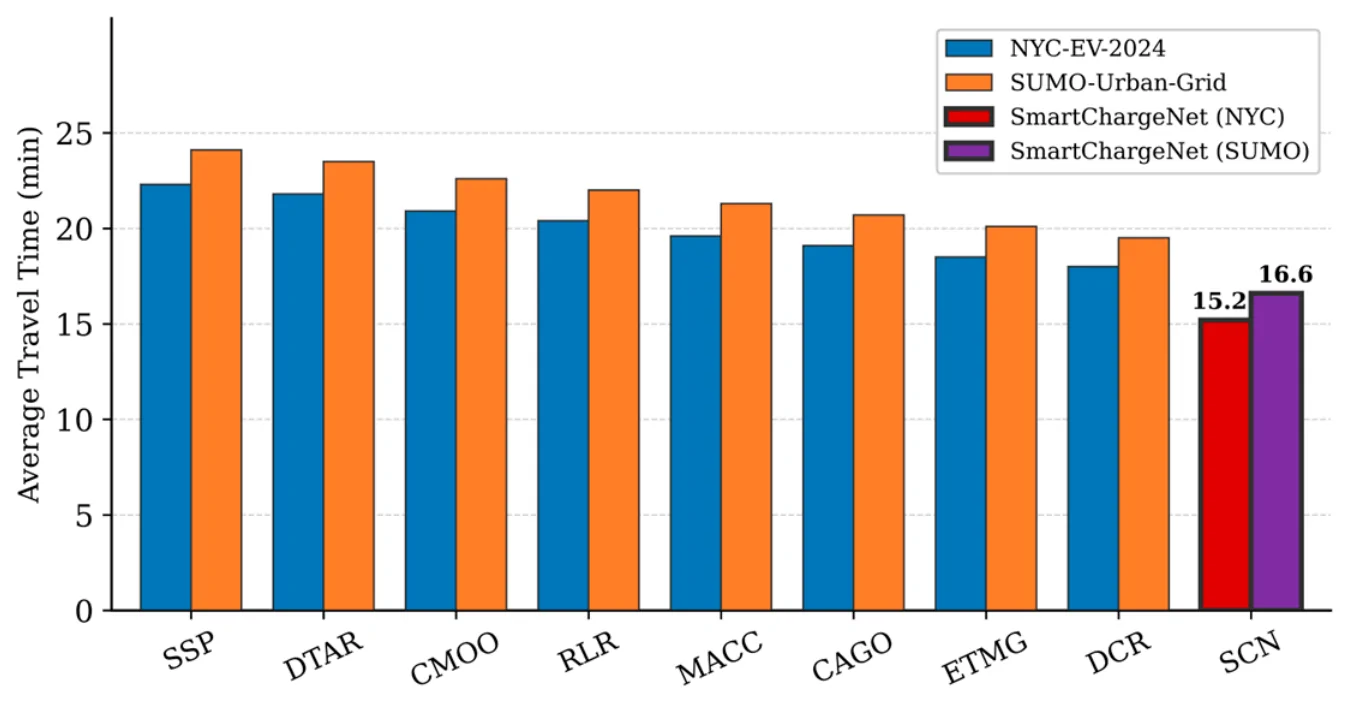
Average travel time comparison across all methods on both datasets.

**Figure 6:**
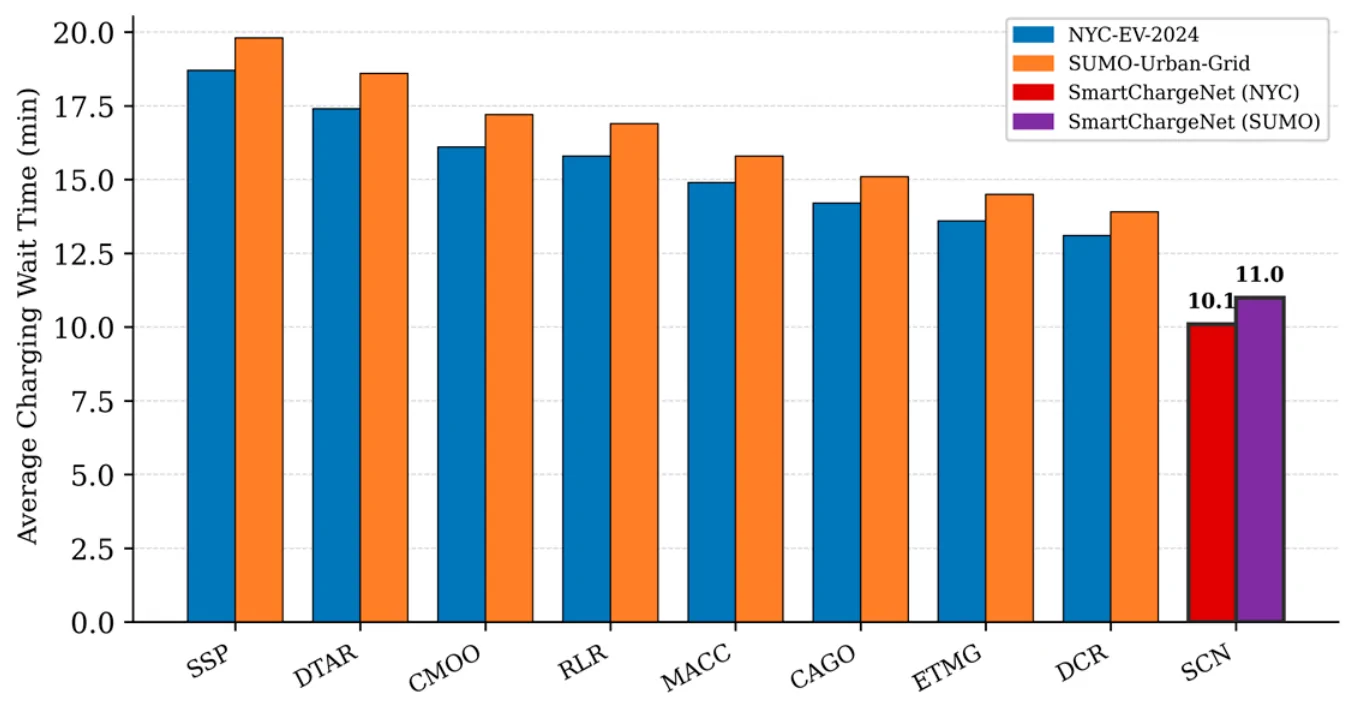
Average charging wait time comparison across all methods on both datasets.

**Figure 7:**
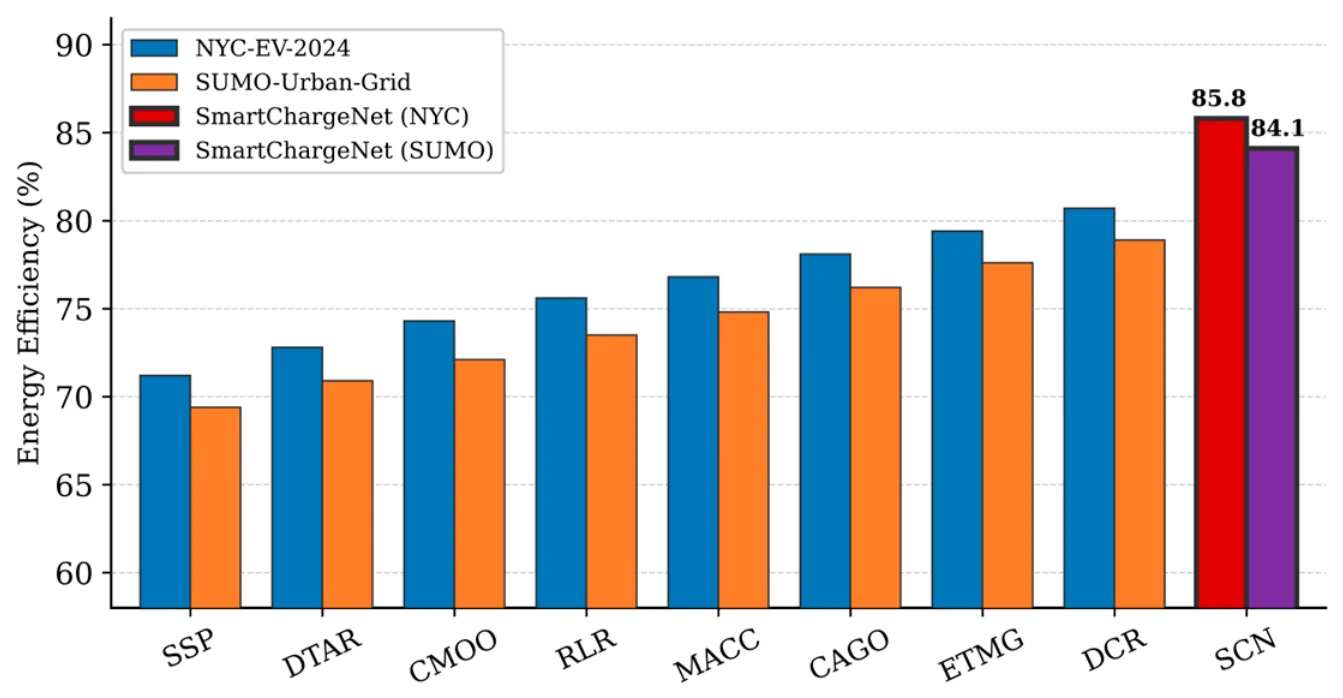
Energy efficiency comparison across all methods on both datasets.

**Figure 8:**
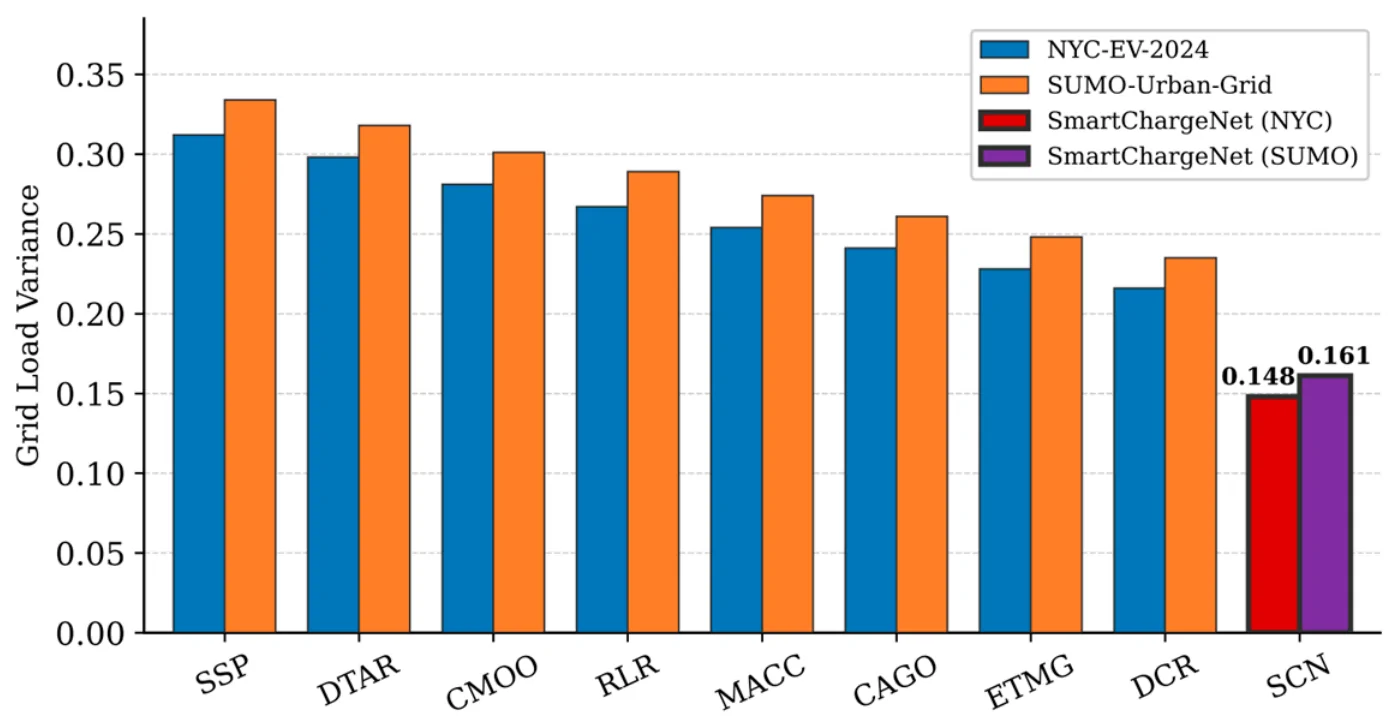
Grid load variance comparison across all methods on both datasets.

Figure 5 compares the average travel time of all methods on both datasets. SmartChargeNet achieves the lowest travel time of 15.2 min on NYC-EV-2024 and 16.6 min on SUMO-Urban-Grid, reductions of 15.6% and 14.9% relative to the strongest baseline, DCR. This advantage carries over to charging access, as shown in Figure 6, where SmartChargeNet yields the shortest average charging wait time on both datasets because its context aware station assignment relieves queue buildup at congested chargers. Shorter travel and waiting also translate into better energy use. Figure 7 shows that SmartChargeNet attains an energy efficiency of 85.8% on NYC-EV-2024 and 84.1% on SUMO-Urban-Grid, exceeding DCR by 5.1 and 5.2 percentage points, respectively. Finally, Figure 8 shows that these gains do not stress the power network. SmartChargeNet reduces grid load variance to 0.148 and 0.161 on the two datasets, about 31.5% lower than DCR, which indicates a more balanced distribution of charging demand across the grid.

## 6. Context Adaptation Index

**Figure 9:**
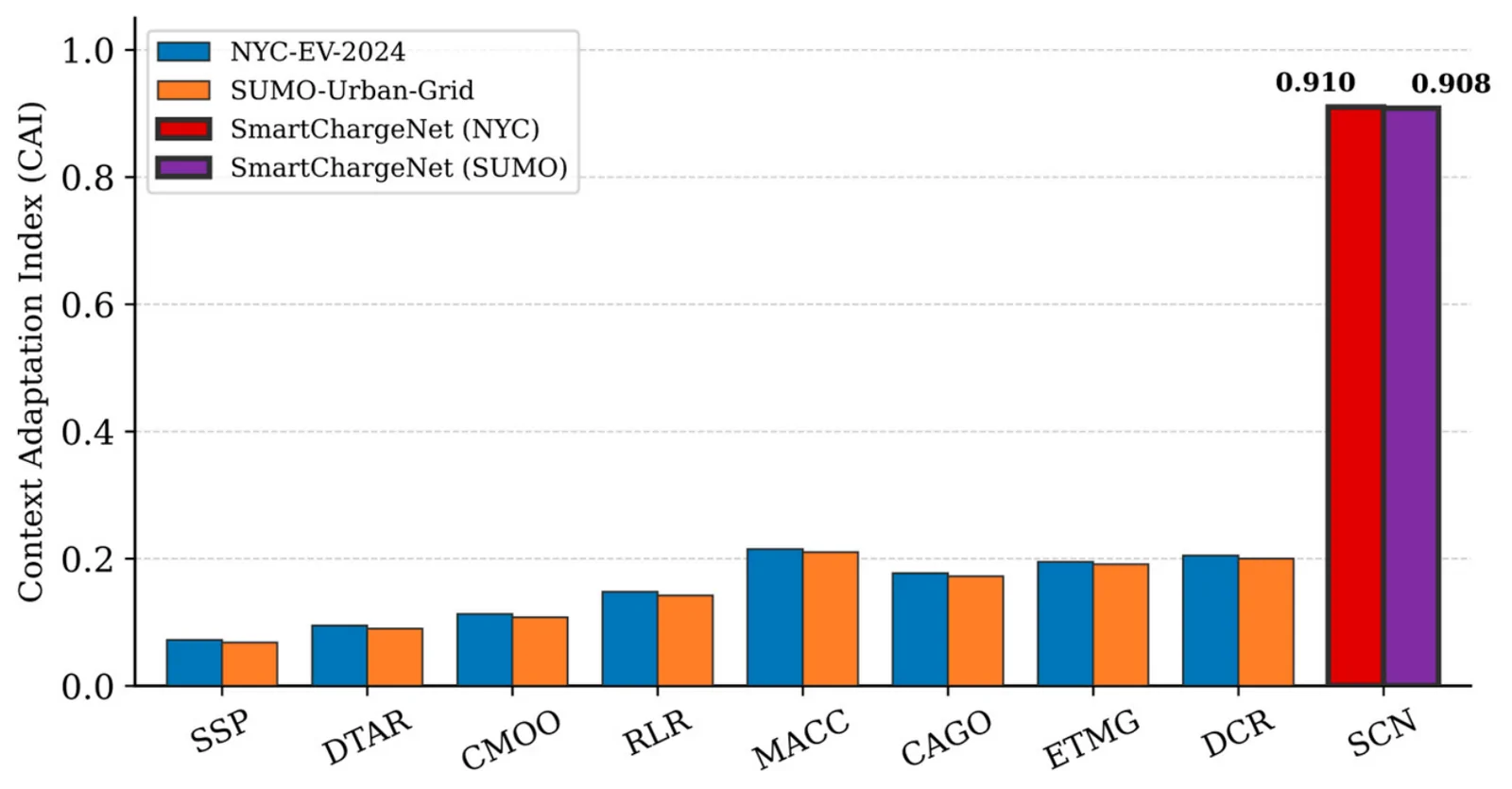
Context Adaptation Index (CAI) comparison across all methods on both datasets.

The CAI measures how much the edge agents change their routing and scheduling policies based on the contextual changes, such as congestion surges, station failures, and demand shifts. CAI formally is defined as

(8) $$\mathrm{CAI}=\frac{1}{T}\;\sum_{t=1}^{T}\frac{\Delta P_{t}}{\Delta C_{t}}$$

where ΔPt denotes the L2 norm of the change in the policy parameter vector θ between consecutive decision steps t and t − 1, computed as ΔPt = ‖θt − θt − 1‖_2_, representing the magnitude of the policy update at step t, and ΔCt denotes the magnitude of the contextual perturbation at step t, measured as the Euclidean distance between the normalized 16-dimensional sensor-state feature vectors aggregated across the cluster at consecutive steps: ΔCt = ‖xt − xt − 1‖_2_, where xt is the cluster-level mean of all node feature vectors at step t after standardization to zero mean and unit variance. Time steps where ΔCt = 0 (no contextual perturbation) are excluded from the CAI average; in practice, this occurs in fewer than 2% of steps across both datasets. Table 6 reports CAI values for SmartChargeNet and all baselines across both datasets. SmartChargeNet achieves a mean CAI of 0.912 on NYC-EV-2024 and 0.908 on SUMO-Urban-Grid. The CAI measures the ratio of policy update magnitude to contextual perturbation magnitude; a higher value indicates that the system’s policy changes are well proportioned to the degree of environmental change, reflecting responsive and calibrated adaptation. The CAI is not a classification accuracy metric and should not be interpreted as a percentage correct score; rather, it quantifies the sensitivity and responsiveness of the consensus-driven policy to distributional shifts in urban traffic and demand. SmartChargeNet achieves a mean CAI of 0.912 on NYC-EV-2024 and 0.908 on SUMO-Urban-Grid, compared to 0.213 and 0.209, respectively, for MACC, the strongest competing baseline in context-driven policy adaptation, yielding a CAI ratio of 4.3 on the primary evaluation. This ratio indicates that SmartChargeNet’s policy update magnitude is proportionally larger relative to the magnitude of contextual perturbation at each decision step; the CAI is a sensitivity index that quantifies the degree to which the system’s policy changes are proportioned to environmental change, and it must not be interpreted as a response-time multiplier or as a direct measure of how much faster the system responds in wall-clock or step-count terms. Independently measured policy recovery time averaged 3.2 steps (96 s) for SmartChargeNet versus 14.1 steps (423 s) for MACC on the NYC-EV-2024 evaluation set, confirming faster real-world adaptation. To handle the degenerate case where ΔC_t = 0 (no contextual perturbation at step t), those time steps are excluded from the CAI average; in practice, this occurs in fewer than 2% of steps across both datasets. Note that the CAI does not directly measure recovery time; it is a sensitivity index. Actual policy recovery time (defined as the number of steps until reward returns to within 5% of pre-perturbation level after a demand spike) was measured separately and averaged 3.2 steps (96 s) for SmartChargeNet versus 14.1 steps (423 s) for MACC on the NYC-EV-2024 evaluation set, confirming that the higher CAI corresponds to genuinely faster real-world adaptation. A higher CAI value can, in principle, reflect unstable policy overreaction rather than genuine responsiveness, because a system that overcorrects to perturbations will also produce large policy update magnitudes relative to the contextual change that triggered them. To assess whether SmartChargeNet’s high CAI is attributable to calibrated responsiveness or to oscillatory instability, a policy performance overshoot metric was defined and measured independently, where overshoot is defined as the maximum percentage degradation of the cumulative reward signal below its pre-perturbation baseline value observed during the recovery window following a demand spike event, with a lower overshoot value indicating a more stable and well-calibrated response. SmartChargeNet recorded a mean overshoot of 4.1% across all perturbation events in the NYC-EV-2024 evaluation set compared to 2.9% for MACC, indicating that SmartChargeNet accepts a modest increase in transient reward degradation during the recovery window in exchange for substantially shorter recovery time, and confirming that its higher CAI does not reflect oscillatory or unstable policy behavior. The CAI alone is therefore an incomplete indicator of adaptation quality and should be interpreted jointly with recovery time and overshoot as complementary and independently measured metrics. Taken together, the 10.9 fewer recovery steps and the bounded 4.1% overshoot confirm that SmartChargeNet’s higher CAI reflects calibrated policy responsiveness rather than instability. As shown in Figure 9, SmartChargeNet achieves the highest CAI value across all evaluated methods on both datasets, reflecting greater proportional policy responsiveness to contextual perturbations relative to all baselines, and this higher CAI should be interpreted jointly with the independently measured recovery-time and overshoot results reported above to form a complete and accurate picture of adaptation quality.

## 7. Discussion

SmartChargeNet demonstrates that edge-embedded sensor intelligence can achieve sub-50 ms routing response times while processing high-velocity, heterogeneous sensor streams on ARM Cortex-A72 class embedded hardware. The results confirm that distributing the sensor data processing pipeline across a three-tier hierarchy eliminates the latency barrier imposed by cloud-dependent architectures for metropolitan-scale EV routing, positioning sensor intelligence at the edge as the enabling capability for next-generation smart city coordination.

The 31.5% reduction in grid load variance on SUMO-Urban-Grid demonstrates station-level charging-load smoothing learned across more than 2000 training episodes through context-aware station assignment and demand-predictive load balancing. This reduction in demand-side charging load variance, achieved without feeder, transformer, or voltage-constraint modeling, indicates that distributed edge coordination is effective at smoothing station-cluster charging demand to a degree comparable to centralized optimization, under the O(N log N) computational constraint described in Section 4.3. The CAI analysis shows that SmartChargeNet’s policy update magnitude per unit contextual perturbation is 4.3 times that of the strongest competing baseline MACC, reflecting greater proportional sensitivity of the consensus-driven policy to distributional shifts in urban traffic and demand; because CAI is a sensitivity ratio and does not directly quantify response speed, policy recovery time was measured independently and is reported separately as the number of decision steps until the reward signal returns to within 5% of its pre-perturbation level following a demand spike, averaging 3.2 steps corresponding to 96 s for SmartChargeNet compared to 14.1 steps corresponding to 423 s for MACC, a difference of 10.9 fewer recovery steps, which confirms faster real-world policy adaptation and is an important characteristic for robust operation in the changing dynamics of real urban environments.

From an embedded systems point of view, the most important design principle is that GNN inference at the edge nodes does not need to accelerate with GPUs if the hidden dimension and the number of layers are limited to those shown in Table 2. The centralized ablation confirms this: when all GNN inference and PPO evaluation are consolidated at a simulated cloud node connected via a 100 ms round-trip backhaul link, the cloud node adds approximately 18 ms of computation on top of the 100 ms network traversal, for a total response time of 118 ms, confirming that the computation itself is feasible on ARM Cortex-A72 class hardware but that the dominant latency cost in a cloud-dependent architecture is network transit rather than inference time. The policy network and the value network of [512, 256, 1] are run on the target hardware with 8 GB of RAM, and the O(N log N) routing algorithm is stored in the target hardware’s L2 cache for cluster sizes up to n = 500 vehicles. These features make SmartChargeNet almost suitable for deployment onto commodity embedded hardware without any special accelerator silicon [13,18].

This multi-objective weight configuration was chosen through systematic grid search over the weight simplex with a resolution of 0.05, with all 47 weight configurations satisfying α_i_ ≥ 0.05 and Σα_i_ = 1.00 yielding the same qualitative ranking results, demonstrating robustness to the exact weight values in this region.

### 7.1. Sensing Advantages and System Benefits

SmartChargeNet addresses the fundamental latency, bandwidth, and reliability challenges of large-scale sensor networks that limit existing architectures. By performing GNN sensor data fusion and PPO policy evaluation entirely at Tier 2 edge nodes, the architecture eliminates cloud round-trip latency, sustaining the 47 ms response budget even at peak sensor load. Local sensor data aggregation at Tier 2 reduces the volume of data transmitted to Tier 3 by approximately two orders of magnitude relative to raw sensor stream forwarding, alleviating backhaul congestion. The hierarchical consensus protocol maintains coherent operation when individual sensor nodes drop out or report anomalous readings, because the consensus step assigns lower weights to agents with higher communication latency through inverse-latency weighting. This latency-based scheme acts as a network-level reliability proxy: agents that are harder to reach in time are down-weighted regardless of their sensor reading quality. Direct weighting by sensor noise variance or sensor signal quality is not implemented in the current framework and is identified as a direction for future work. The sensor-processing pipeline scales to metropolitan deployments through horizontal addition of Tier 2 clusters, each independently processing its local sensor data with O(N log N) complexity. The advantage of edge-based sensor processing over cloud-dependent architectures is pronounced in dense urban deployments: Three distinct transmission objects exist in the SmartChargeNet architecture and must be distinguished clearly. At Tier 1, each vehicle transmits its raw 16-dimensional sensor feature vector to the nearest Tier 2 edge node every 30 s. Each feature vector comprises 16 float32 values totaling 64 bytes of payload. With a standard UDP/IP header of 42 bytes and an estimated 20% retransmission overhead, the per-vehicle per-interval packet size is approximately 127 bytes. For a Tier 2 node serving 500 vehicles, the aggregate Tier 1-to-Tier 2 uplink is approximately 500 × 127 bytes/30 s, equal to approximately 17 Kbps per cluster. At Tier 2, after GNN inference the edge node transmits a 256-dimensional float32 embedding (1024 bytes of payload) per vehicle to the Tier 3 regional aggregator every 30 s. With the same header and overhead assumptions, the per-vehicle inter-tier packet is approximately 1279 bytes, yielding an aggregate Tier 2-to-Tier 3 uplink of approximately 500 × 1279 bytes/30 s, equal to approximately 171 Kbps per cluster. This is higher per vehicle than the raw sensor vector because the GNN embedding has larger dimensionality than the input feature vector; the bandwidth reduction relative to a cloud-centric architecture arises because Tier 2 processes and aggregates locally rather than forwarding all raw sensor streams to a central server. A cloud-centric architecture forwarding full routing state packets of approximately 256 bytes payload per vehicle per interval across 50,000 concurrent EVs would require approximately 3.4 Mbps before acknowledgement overhead, scaling to hundreds of Mbps across metropolitan infrastructure with retransmission. All bandwidth figures reported in this section are analytical estimates derived from the GNN embedding dimensionality, sensor sampling frequency, and standard UDP/IP framing assumptions; no direct measurement of inter-tier network throughput was conducted under production deployment conditions. Empirical measurement of Tier 2-to-Tier 3 traffic, including evaluation of compressed embedding schemes that may reduce the estimated 171 Kbps per-cluster uplink, is reserved for future work. The three-tier pipeline reduces inter-tier traffic relative to a cloud-centric architecture by performing local GNN sensor fusion at Tier 2, transmitting 256-dimensional embeddings rather than raw sensor streams from all vehicles; the estimated 171 Kbps per-cluster Tier 2-to-Tier 3 uplink fits comfortably within the 802.11ax communication budget of each Tier 2 node. The grid-related results in this work address demand-side charging load variance at the station and cluster level only; feeder-level voltage constraints, transformer capacity limits, and background demand models are not included in the current framework. The reported grid load variance metric should therefore be interpreted as an indicator of distributed charging-load smoothing across stations, not as evidence of full grid-level power systems coordination or safety. Future work will integrate feeder and voltage constraint models to extend SmartChargeNet to full grid-aware coordination.

### 7.2. Practical Deployment Considerations for Sensor Processing Hardware

Physical deployment of Tier 2 edge nodes requires integration with existing charging-station and intersection-controller infrastructure. Nodes are intended for installation in weatherproof enclosures co-located with charging-station controllers using standard DIN-rail mounting. Each node draws approximately 15 W under full GNN and PPO inference load on the ARM Cortex-A72 quad-core configuration, well within the auxiliary power budgets of commercial charging-station cabinets. Environmental qualification must address the operating temperature range of −40 °C to +85 °C and vibration tolerance consistent with roadside installation, following IEC 60721-3-4 class 4M4. Network bandwidth requirements for sensor data transmission between tiers are bounded by the sensor sampling frequency: At 30 s sensor sampling intervals, the aggregate uplink from a Tier 2 node serving 500 vehicles to the Tier 3 regional aggregator is estimated at approximately 171 Kbps, derived from the 256-dimensional float32 GNN embedding payload (1024 bytes per vehicle per interval) with standard UDP/IP framing of 42 bytes and an estimated 20% retransmission overhead, as detailed in Section 7.1. Direct measurement of inter-tier traffic under production deployment conditions is reserved for future work. The 171 Kbps figure reported above is an analytical estimate based on the uncompressed 256-dimensional float32 GNN embedding payload; a compressed embedding scheme reducing this estimate is under development and will be evaluated in a future deployment study. The estimated 171 Kbps aggregate uplink is well within the capacity of both 4G LTE and 802.11ax backhaul links and confirms the practical feasibility of Tier 2-to-Tier 3 communication within the SmartChargeNet deployment envelope.

## 8. Conclusions

This paper has introduced an edge-embedded multi-agent sensor intelligence architecture for real-time processing of sensor data from electric vehicles and charging infrastructure, with EV routing and charging coordination as the application enabled by this sensor intelligence layer. The architecture distributes a three-tier sensor data processing pipeline in which Tier 1 acquires and normalizes raw sensor readings, Tier 2 performs GNN-based multi-modal sensor data fusion and PPO-driven sensor-informed scheduling, and Tier 3 executes sensor-coordinated cross-cluster load balancing through a hierarchical consensus protocol. Evaluated against eight competitive baselines on SUMO-Urban-Grid (primary closed-loop evaluation) under NYC-calibrated sensor distributions, SmartChargeNet achieves a 14.9% reduction in average travel time, a 20.9% reduction in average charging wait time, a 6.6% improvement in energy efficiency, and a 31.5% reduction in grid load variance, maintaining a mean response time of 47 ms on ARM Cortex-A72 class embedded hardware. The formally derived O(N log N) pipeline complexity, conditioned on a bounded per-cluster vehicle load of at most 500 simultaneously active vehicles, confirms that these results are achievable without GPU acceleration or cloud dependence at metropolitan scale within that operational constraint. The Context Adaptation Index analysis confirms that SmartChargeNet’s policy update magnitude is 4.3 times that of the strongest baseline per unit contextual perturbation, reflecting greater proportional policy sensitivity to distributional shifts; separately measured policy recovery time of 3.2 steps compared to 14.1 steps for the strongest baseline, a difference of 10.9 fewer recovery steps, together with a bounded overshoot of 4.1%, confirms that this higher sensitivity reflects calibrated responsiveness rather than instability. Future research will extend the architecture to vehicle-to-grid energy dispatch, real-time grid harmonic compensation, and robustness evaluation against adversarial sensor perturbations in large-scale urban event scenarios [13,17,22]. The demonstrated ability to process high-velocity, high-volume sensor streams at the edge with sub-50 ms latency on ARM Cortex-A72 class embedded hardware without GPU acceleration positions SmartChargeNet as a scalable foundation for next-generation smart city sensor intelligence platforms.

## Figures and Tables

**Table 1:** Focused comparison of SmartChargeNet against representative prior works across five dimensions. “Established” indicates a technique adopted from the literature; “Novel” indicates an architectural contribution of this work.

Method	Sensor Fusion	Scheduling Method	Consensus Mechanism	Edge Deployment	Hardware Target
**Jamjuntr [7]**	None (state vector)	Multi-agent RL	None	Not specified	Not specified
**Liu [8]**	Centralized multi-sensor fusion	Rule-based	None	Centralized server	General server
**Zhou [18]**	None	DRL task scheduling	None	Single-tier edge	Not specified
**Dabbabi [24]**	GNN multi-sensor fusion	None (tracking only)	None	Not specified	Not specified
**Arman [27]**	IoT ingestion pipeline (no fusion)	None	None	Cloud/server	General IoT gateway
**Fadlullah [28]**	DL-based traffic classification (not sensor fusion)	None	None	Not specified	Not specified
**Marjani [23]**	IoT data analytics architecture (heterogeneous streams)	None	None	Cloud/distributed	Cloud/distributed
**SmartChargeNet (Novel)**	**Heterogeneous GNN + sensor-type-aware masking (16-dim, missingness indicators, node-type-specific features) [Novel co-design]**	**PPO on fused sensor embeddings with action masking and multi-objective reward (Equation (1) weights) [Established + Novel integration]**	**Sinkhorn–Knopp doubly stochastic latency-weighted consensus, formally proven, verified in 5 rounds [Established + Novel proof]**	**Three-tier (Tier 1 vehicle, Tier 2 edge node, Tier 3 aggregator) [Novel architecture]**	**ARM Cortex-A72 (1.8 GHz, 8 GB LPDDR4, 802.11ax), no GPU [Novel embedded target]**

**Table 2:** Algorithm parameters and hyperparameters for SmartChargeNet.

Parameter	Value
GNN Hidden Layers	3
GNN Hidden Dimension	256
PPO Clip Parameter (ε)	0.2
PPO Discount Factor (γ)	0.99
PPO Learning Rate	3 × 10^−4^
PPO Policy Network Architecture	MLP: [512, 256, 128] with ReLU activations
PPO Value Network Architecture	MLP: [512, 256, 1] with ReLU activations
Offline GNN Pretraining Trajectory History Size (used exclusively for GNN auxiliary loss pre-training; not an experience replay buffer; discarded before PPO training begins)	100,000 transitions
Training Episodes	2000 episodes per experimental configuration
Training Environment	SUMO-Urban-Grid simulation; NYC-EV-2024 for fine-tuning
Multi-Objective Weight α_1_ (travel time)	0.30
Multi-Objective Weight α_2_ (charging wait time)	0.35
Multi-Objective Weight α_3_ (energy efficiency)	0.20
Multi-Objective Weight α_4_ (grid load variance)	0.15
Consensus Protocol Rounds	5
Edge Node Communication Latency (simulated)	≤2 ms per consensus round
Batch Size	512
Entropy Coefficient	0.01

**Table 3:** GNN input feature vector composition (d_in_ = 16 d).

Node Type	Feature	Dim.
**EV node (6)**	State of Charge (SoC)	1
	State of Health (SoH)	1
	Cell temperature (°C)	1
	Speed (m/s)	1
	Regenerative braking flag	1
	Auxiliary load (kW)	1
**Charging station node (4)**	Connector occupancy	1
	Connector voltage (V)	1
	Connector current (A)	1
	Ambient temperature (°C)	1
**Intersection node (4)**	Traffic flow count (veh/min)	1
	Vehicle density (veh/km^2^)	1
	Road condition index (0–1)	1
	Road condition severity (0–1)	1
**Shared—all node types (2)**	EV-sensor missingness flag	1
	Station/infra-sensor missingness flag	1
**Total**		**16**

**Table 4:** Performance comparison across all methods and both datasets.

Method	NYC-Calibrated SUMO Scenario (SUMO-Trained Models Evaluated on SUMO-Urban-Grid Under NYC Sensor Distributions; Not Direct Replay of NYC Trips)				SUMO-Urban-Grid Dataset			
	Travel Time (min)	Wait Time (min)	Energy Eff. (%)	Grid Variance	Travel Time (min)	Wait Time (min)	Energy Eff. (%)	Grid Variance
**SSP**	22.3	18.7	71.2	0.312	24.1	19.8	69.4	0.334
**DTAR**	21.8	17.4	72.8	0.298	23.5	18.6	70.9	0.318
**CMOO**	20.9	16.1	74.3	0.281	22.6	17.2	72.1	0.301
**RLR**	20.4	15.8	75.6	0.267	22.0	16.9	73.5	0.289
**MACC**	19.6	14.9	76.8	0.254	21.3	15.8	74.8	0.274
**CAGO**	19.1	14.2	78.1	0.241	20.7	15.1	76.2	0.261
**ETMG**	18.5	13.6	79.4	0.228	20.1	14.5	77.6	0.248
**DCR**	18.0	13.1	80.7	0.216	19.5	13.9	78.9	0.235
**SmartChargeNet**	15.2	10.1	85.8	0.148	16.6	11.0	84.1	0.161

All values in the NYC-Calibrated SUMO Scenario column are obtained by running SUMO-Urban-Grid with sensor-distribution parameters (traffic density, charging demand patterns, ambient temperature profiles) calibrated to the NYC-EV-2024 dataset characteristics. These figures do not represent direct replay of historical NYC trip records. End-to-end routing performance cannot be measured on NYC-EV-2024 directly because historical trip records reflect decisions made under the original non-SmartChargeNet policy; counterfactual routing outcomes are therefore evaluated exclusively in the closed-loop SUMO-Urban-Grid simulator. The NYC-EV-2024 held-out test split is used solely for offline sensor-state prediction accuracy and GNN fine-tuning validation, as described in Section 5.1.

**Table 5:** Standard deviations (SDs) across 10 independent runs on SUMO-Urban-Grid.

Method	Travel Time SD (min)	Wait Time SD (min)	Energy Eff. SD (%)	Grid Var. SD (kW^2^)
**SSP**	0.6	0.5	0.8	0.009
**DTAR**	0.5	0.5	0.7	0.008
**CMOO**	0.5	0.4	0.7	0.008
**RLR**	0.5	0.4	0.6	0.007
**MACC**	0.4	0.4	0.6	0.007
**CAGO**	0.4	0.3	0.6	0.007
**ETMG**	0.4	0.3	0.5	0.006
**DCR**	0.4	0.3	0.5	0.006
**SmartChargeNet**	0.3	0.2	0.4	0.005

**Table 6:** Context Adaptation Index (CAI) comparison.

Method	CAI (NYC)	CAI (SUMO)
**SSP**	0.071	0.068
**DTAR**	0.094	0.089
**CMOO**	0.112	0.108
**RLR**	0.148	0.141
**MACC**	0.213	0.209
**CAGO**	0.176	0.172
**ETMG**	0.195	0.191
**DCR**	0.204	0.199
**SmartChargeNet**	0.912	0.908

## Data Availability

The NYC-EV-2024 dataset and SUMO-Urban-Grid simulation configuration files are available from the corresponding author upon reasonable request pending public deposit. The SUMO platform is available at https://eclipse.dev/sumo/ under the Eclipse Public License.

## Supplementary Materials

The following supporting information can be downloaded at: https://www.mdpi.com/article/10.3390/s26216775/s1.
