# Supplementary material for: SmartChargeNet: An Edge-Embedded Multi-Agent Sensor Intelligence Architecture for Real-Time Electric Vehicle Routing and Charging Infrastructure Coordination in Smart Urban Grids
Source: Sensors (Basel). 2026 Oct 26;26(21):6775. doi: 10.3390/s26216775 (PMC13765661; doi:10.3390/s26216775)
Supplement: Supplementary file 1 [file sensors-26-06775-s001.zip › sensors-4575951-supplementary.pdf]

Supplementary Material

SmartChargeNet: An Edge-Embedded Multi-Agent Sensor Intelligence Architecture for Real-Time Electric Vehicle Routing and Charging Infrastructure Coordination in Smart Urban Grids

Abdulaziz H. Alshehri [1,2]

1 Department of Civil Engineering, College of Engineering, Najran University, Najran 66462, Saudi Arabia; ahalshehry@nu.edu.sa
2 Science and Engineering Research Center, Najran University, Najran 66462, Saudi Arabia

This document contains Tables S1 to S5 and Sections S1 to S3 referenced in the main text. Two supplementary data files accompany this document: RewardLog_AllSeeds.csv (raw per-episode PPO reward records for all ten seeds, referenced in Section 4.2) and NYC_EV_2024_plate_class_map.csv (SHA-256 hashed plate-to-class mapping produced by the linking procedure of Section 5.1).

All configurations satisfy $\alpha1$, $\alpha2$, $\alpha3$, $\alpha4 \geq 0.05$ and $\Sigma\alpha i = 1.00$ at a grid resolution of 0.05. Rank columns give the SmartChargeNet rank among the nine compared methods for travel time / wait time / energy efficiency / grid load variance (1 = best). Reductions relative to DCR are measured on the SUMO-Urban-Grid held-out test split. Configuration 1 is the baseline used for all results in the main text; configurations 35 and 42 are the travel-time-favoring and grid-variance-favoring configurations quoted in Section 3.2.

Table S1. Multi-objective weight sensitivity analysis: all 47 weight configurations.

No.	Label	$\alpha1$	$\alpha2$	$\alpha3$	$\alpha4$	Rank (NYC-calibrated SUMO)	Rank (SUMO-Urban-Grid)	Travel time reduction vs DCR (%)	Grid variance reduction vs DCR (%)
1	Baseline	0.30	0.35	0.20	0.15	1 / 1 / 1 / 1	1 / 1 / 1 / 1	14.9	31.5
2	T to W (0.05)	0.25	0.40	0.20	0.15	1 / 1 / 1 / 1	1 / 1 / 1 / 1	14.7	31.5
3	T to E (0.05)	0.25	0.35	0.25	0.15	1 / 1 / 1 / 1	1 / 1 / 1 / 1	14.7	31.5
4	T to V (0.05)	0.25	0.35	0.20	0.20	1 / 1 / 1 / 1	1 / 1 / 1 / 1	14.7	31.8
5	W to T (0.05)	0.35	0.30	0.20	0.15	1 / 1 / 1 / 1	1 / 1 / 1 / 1	14.9	31.5
6	W to E (0.05)	0.30	0.30	0.25	0.15	1 / 1 / 1 / 1	1 / 1 / 1 / 1	14.9	31.5
7	W to V (0.05)	0.30	0.30	0.20	0.20	1 / 1 / 1 / 1	1 / 1 / 1 / 1	14.9	31.8
8	E to T (0.05)	0.35	0.35	0.15	0.15	1 / 1 / 1 / 1	1 / 1 / 1 / 1	14.9	31.5
9	E to W (0.05)	0.30	0.40	0.15	0.15	1 / 1 / 1 / 1	1 / 1 / 1 / 1	14.9	31.5
10	E to V (0.05)	0.30	0.35	0.15	0.20	1 / 1 / 1 / 1	1 / 1 / 1 / 1	14.9	31.8
11	V to T (0.05)	0.35	0.35	0.20	0.10	1 / 1 / 1 / 1	1 / 1 / 1 / 1	14.9	31.2
12	V to W (0.05)	0.30	0.40	0.20	0.10	1 / 1 / 1 / 1	1 / 1 / 1 / 1	14.9	31.2
13	V to E (0.05)	0.30	0.35	0.25	0.10	1 / 1 / 1 / 1	1 / 1 / 1 / 1	14.9	31.2
14	T to W (0.10)	0.20	0.45	0.20	0.15	1 / 1 / 1 / 1	1 / 1 / 1 / 1	14.5	31.5
15	T to E (0.10)	0.20	0.35	0.30	0.15	1 / 1 / 1 / 1	1 / 1 / 1 / 1	14.5	31.5

16	T to V (0.10)	0.20	0.35	0.20	0.25	1 / 1 / 1 / 1	1 / 1 / 1 / 1	14.5	32.1
17	W to T (0.10)	0.40	0.25	0.20	0.15	1 / 1 / 1 / 1	1 / 1 / 1 / 1	14.9	31.5
18	W to E (0.10)	0.30	0.25	0.30	0.15	1 / 1 / 1 / 1	1 / 1 / 1 / 1	14.9	31.5
19	W to V (0.10)	0.30	0.25	0.20	0.25	1 / 1 / 1 / 1	1 / 1 / 1 / 1	14.9	32.1
20	E to T (0.10)	0.40	0.35	0.10	0.15	1 / 1 / 1 / 1	1 / 1 / 1 / 1	14.9	31.5
21	E to W (0.10)	0.30	0.45	0.10	0.15	1 / 1 / 1 / 1	1 / 1 / 1 / 1	14.9	31.5
22	E to V (0.10)	0.30	0.35	0.10	0.25	1 / 1 / 1 / 1	1 / 1 / 1 / 1	14.9	32.1
23	V to T (0.10)	0.40	0.35	0.20	0.05	1 / 1 / 1 / 1	1 / 1 / 1 / 1	14.9	30.9
24	V to W (0.10)	0.30	0.45	0.20	0.05	1 / 1 / 1 / 1	1 / 1 / 1 / 1	14.9	30.9
25	V to E (0.10)	0.30	0.35	0.30	0.05	1 / 1 / 1 / 1	1 / 1 / 1 / 1	14.9	30.9
26	T to W (0.15)	0.15	0.50	0.20	0.15	1 / 1 / 1 / 1	1 / 1 / 1 / 1	14.3	31.5
27	T to E (0.15)	0.15	0.35	0.35	0.15	1 / 1 / 1 / 1	1 / 1 / 1 / 1	14.3	31.5
28	T to V (0.15)	0.15	0.35	0.20	0.30	1 / 1 / 1 / 1	1 / 1 / 1 / 1	14.3	32.5
29	W to T (0.15)	0.45	0.20	0.20	0.15	1 / 1 / 1 / 1	1 / 1 / 1 / 1	14.9	31.5
30	W to E (0.15)	0.30	0.20	0.35	0.15	1 / 1 / 1 / 1	1 / 1 / 1 / 1	14.9	31.5
31	W to V (0.15)	0.30	0.20	0.20	0.30	1 / 1 / 1 / 1	1 / 1 / 1 / 1	14.9	32.5
32	E to T (0.15)	0.45	0.35	0.05	0.15	1 / 1 / 1 / 1	1 / 1 / 1 / 1	14.9	31.5
33	E to W (0.15)	0.30	0.50	0.05	0.15	1 / 1 / 1 / 1	1 / 1 / 1 / 1	14.9	31.5
34	E to V (0.15)	0.30	0.35	0.05	0.30	1 / 1 / 1 / 1	1 / 1 / 1 / 1	14.9	32.5
35	Travel emphasis 1	0.45	0.30	0.15	0.10	1 / 1 / 1 / 1	1 / 1 / 1 / 1	14.9	31.2
36	Travel emphasis 2	0.50	0.25	0.15	0.10	1 / 1 / 1 / 1	1 / 1 / 1 / 1	14.9	31.2
37	Wait emphasis 1	0.25	0.50	0.15	0.10	1 / 1 / 1 / 1	1 / 1 / 1 / 1	14.7	31.2
38	Wait emphasis 2	0.20	0.55	0.15	0.10	1 / 1 / 1 / 1	1 / 1 / 1 / 1	14.5	31.2
39	Energy emphasis 1	0.25	0.30	0.35	0.10	1 / 1 / 1 / 1	1 / 1 / 1 / 1	14.7	31.2
40	Energy emphasis 2	0.20	0.25	0.45	0.10	1 / 1 / 1 / 1	1 / 1 / 1 / 1	14.5	31.2
41	Grid emphasis 1	0.25	0.30	0.15	0.30	1 / 1 / 1 / 1	1 / 1 / 1 / 1	14.7	32.5
42	Grid emphasis 2	0.20	0.25	0.15	0.40	1 / 1 / 1 / 1	1 / 1 / 1 / 1	14.5	33.1
43	Uniform	0.25	0.25	0.25	0.25	1 / 1 / 1 / 1	1 / 1 / 1 / 1	14.7	32.1
44	Travel and wait emphasis	0.40	0.40	0.10	0.10	1 / 1 / 1 / 1	1 / 1 / 1 / 1	14.9	31.2
45	Mixed 1	0.35	0.40	0.10	0.15	1 / 1 / 1 / 1	1 / 1 / 1 / 1	14.9	31.5
46	Mixed 2	0.25	0.40	0.25	0.10	1 / 1 / 1 / 1	1 / 1 / 1 / 1	14.7	31.2
47	Mixed 3	0.40	0.30	0.10	0.20	1 / 1 / 1 / 1	1 / 1 / 1 / 1	14.9	31.8

Normalized policy variance ratio is the mean squared deviation of agent policy parameter vectors from the cluster mean at round k divided by the same quantity at round 0. Convergence is declared when the ratio falls strictly below 0.01. Values are means over 10 independent runs for the three representative cluster topologies (ring, mesh, star) encountered in the SUMO-Urban-Grid and NYC-EV-2024 evaluation environments, with 12 Tier 2 agents per cluster and inverse-latency weights made doubly stochastic by Sinkhorn–Knopp at every round.

Table S2. Hierarchical consensus convergence: normalized policy variance ratio per round.

Consensus round	Ring	Mesh	Star
0	1.000	1.000	1.000
1	0.412	0.231	0.318
2	0.168	0.054	0.104
3	0.071	0.012	0.036
4	0.024	0.003	0.013
5	0.008	0.001	0.005
Round at which ratio < 0.01	5	4	5

Entries are mean ± SD [95% CI], with CI = mean ± t0.975,9 · SD/√10 (t0.975,9 = 2.262). Means correspond to the SUMO-Urban-Grid columns of Table 4 and SDs to Table 5 of the main text.

Table S3. Means, standard deviations, and 95% confidence intervals across 10 independent runs on SUMO-Urban-Grid (primary evaluation).

Method	Travel time (min)	Wait time (min)	Energy efficiency (%)	Grid load variance (kW2)
SSP	24.1 ± 0.6 [23.7, 24.5]	19.8 ± 0.5 [19.4, 20.2]	69.4 ± 0.8 [68.8, 70.0]	0.334 ± 0.009 [0.328, 0.340]
DTAR	23.5 ± 0.5 [23.1, 23.9]	18.6 ± 0.5 [18.2, 19.0]	70.9 ± 0.7 [70.4, 71.4]	0.318 ± 0.008 [0.312, 0.324]
CMOO	22.6 ± 0.5 [22.2, 23.0]	17.2 ± 0.4 [16.9, 17.5]	72.1 ± 0.7 [71.6, 72.6]	0.301 ± 0.008 [0.295, 0.307]
RLR	22.0 ± 0.5 [21.6, 22.4]	16.9 ± 0.4 [16.6, 17.2]	73.5 ± 0.6 [73.1, 73.9]	0.289 ± 0.007 [0.284, 0.294]
MACC	21.3 ± 0.4 [21.0, 21.6]	15.8 ± 0.4 [15.5, 16.1]	74.8 ± 0.6 [74.4, 75.2]	0.274 ± 0.007 [0.269, 0.279]
CAGO	20.7 ± 0.4 [20.4, 21.0]	15.1 ± 0.3 [14.9, 15.3]	76.2 ± 0.6 [75.8, 76.6]	0.261 ± 0.007 [0.256, 0.266]
ETMG	20.1 ± 0.4 [19.8, 20.4]	14.5 ± 0.3 [14.3, 14.7]	77.6 ± 0.5 [77.2, 78.0]	0.248 ± 0.006 [0.244, 0.252]
DCR	19.5 ± 0.4 [19.2, 19.8]	13.9 ± 0.3 [13.7, 14.1]	78.9 ± 0.5 [78.5, 79.3]	0.235 ± 0.006 [0.231, 0.239]
SmartChargeNet	16.6 ± 0.3 [16.4, 16.8]	11.0 ± 0.2 [10.9, 11.1]	84.1 ± 0.4 [83.8, 84.4]	0.161 ± 0.005 [0.157, 0.165]

Station occupancy forecasting and travel-time estimation are the two auxiliary prediction heads of the GNN (Section 4.1). Values are mean ± SD across the 10 independent runs. The fine-tuned model is the SUMO-trained GNN after fine-tuning on the NYC-EV-2024 January to April 2024 split.

Table S3. b. NYC-EV-2024 offline sensor-state prediction statistics (held-out test split, leave-one-out cross-validation within the fine-tuning folds).

Prediction target	Metric	SUMO-trained GNN fine-tuning)	(noSUMO-trained GNN fine-tuned on NYC-EV-2024
Station occupancy (fraction)	MAE	0.142 ± 0.009	0.087 ± 0.006
Station occupancy (fraction)	RMSE	0.198 ± 0.011	0.121 ± 0.008
Station occupancy (fraction)	R2	0.71 ± 0.03	0.89 ± 0.02
Travel time (min)	MAE	2.84 ± 0.17	1.63 ± 0.11
Travel time (min)	RMSE	3.91 ± 0.22	2.27 ± 0.14
Travel time (min)	R2	0.74 ± 0.03	0.91 ± 0.02

Station availability (binary, 30 s horizon)	Accuracy (%)	84.6 ± 1.1	93.2 ± 0.8
Station availability (binary, 30 s horizon)	F1 score	0.81 ± 0.02	0.92 ± 0.01

Two-sided Welch's t-tests comparing SmartChargeNet against each baseline on each of the four primary metrics, n = 10 runs per group. Degrees of freedom follow the Welch–Satterthwaite approximation. Bonferroni-corrected p-values are raw p × 32 (family of 32 tests); the corrected significance threshold is 0.05/32 = 0.0016. Mean difference is SmartChargeNet minus baseline (negative is favorable for travel time, wait time, and grid variance; positive is favorable for energy efficiency). Cohen's d uses the pooled standard deviation $\sqrt{(s1^2 + s2^2)/2}$ and is reported as an absolute value.

Table S4. Complete statistical test results for all 32 pairwise comparisons on SUMO-Urban-Grid.

Metric	Comparison	n	Mean difference	Welch t	df	Raw p	Bonferroni p	95% CI of difference	Cohen's d
Travel time (min)	SmartChargeNet vs SSP	10 / 10	-7.5	-35.36	13.2	1.71e-14	5.48e-13	[-8.0, -7.0]	15.8
Travel time (min)	SmartChargeNet vs DTAR	10 / 10	-6.9	-37.42	14.7	5.08e-16	1.63e-14	[-7.3, -6.5]	16.7
Travel time (min)	SmartChargeNet vs CMOO	10 / 10	-6.0	-32.54	14.7	3.89e-15	1.25e-13	[-6.4, -5.6]	14.6
Travel time (min)	SmartChargeNet vs RLR	10 / 10	-5.4	-29.29	14.7	1.80e-14	5.75e-13	[-5.8, -5.0]	13.1
Travel time (min)	SmartChargeNet vs MACC	10 / 10	-4.7	-29.73	16.7	6.82e-16	2.18e-14	[-5.0, -4.4]	13.3
Travel time (min)	SmartChargeNet vs CAGO	10 / 10	-4.1	-25.93	16.7	6.37e-15	2.04e-13	[-4.4, -3.8]	11.6
Travel time (min)	SmartChargeNet vs ETMG	10 / 10	-3.5	-22.14	16.7	8.32e-14	2.66e-12	[-3.8, -3.2]	9.9
Travel time (min)	SmartChargeNet vs DCR	10 / 10	-2.9	-18.34	16.7	1.71e-12	5.46e-11	[-3.2, -2.6]	8.2
Wait time (min)	SmartChargeNet vs SSP	10 / 10	-8.8	-51.68	11.8	2.79e-15	8.93e-14	[-9.2, -8.4]	23.1
Wait time (min)	SmartChargeNet vs DTAR	10 / 10	-7.6	-44.63	11.8	1.56e-14	5.00e-13	[-8.0, -7.2]	20.0
Wait time (min)	SmartChargeNet vs CMOO	10 / 10	-6.2	-43.84	13.2	1.02e-15	3.25e-14	[-6.5, -5.9]	19.6
Wait time (min)	SmartChargeNet vs RLR	10 / 10	-5.9	-41.72	13.2	1.95e-15	6.24e-14	[-6.2, -5.6]	18.7
Wait time (min)	SmartChargeNet vs MACC	10 / 10	-4.8	-33.94	13.2	2.92e-14	9.35e-13	[-5.1, -4.5]	15.2
Wait time (min)	SmartChargeNet vs CAGO	10 / 10	-4.1	-35.96	15.7	1.72e-16	5.49e-15	[-4.3, -3.9]	16.1

Wait time (min)	SmartChargeNet vs ETMG	10 / 10	-3.5	-30.70	15.7	1.99e-15	6.35e-14	[-3.7, -3.3]	13.7
Wait time (min)	SmartChargeNet vs DCR	10 / 10	-2.9	-25.43	15.7	3.59e-14	1.15e-12	[-3.1, -2.7]	11.4
Energy efficiency (%)	SmartChargeNet vs SSP	10 / 10	14.7	51.97	13.2	1.08e-16	3.46e-15	[14.1, 15.3]	23.2
Energy efficiency (%)	SmartChargeNet vs DTAR	10 / 10	13.2	51.77	14.3	1.10e-17	3.50e-16	[12.7, 13.7]	23.2
Energy efficiency (%)	SmartChargeNet vs CMOO	10 / 10	12.0	47.07	14.3	4.25e-17	1.36e-15	[11.5, 12.5]	21.0
Energy efficiency (%)	SmartChargeNet vs RLR	10 / 10	10.6	46.48	15.7	3.18e-18	1.02e-16	[10.1, 11.1]	20.8
Energy efficiency (%)	SmartChargeNet vs MACC	10 / 10	9.3	40.78	15.7	2.43e-17	7.78e-16	[8.8, 9.8]	18.2
Energy efficiency (%)	SmartChargeNet vs CAGO	10 / 10	7.9	34.64	15.7	3.06e-16	9.79e-15	[7.4, 8.4]	15.5
Energy efficiency (%)	SmartChargeNet vs ETMG	10 / 10	6.5	32.10	17.2	8.95e-17	2.86e-15	[6.1, 6.9]	14.4
Energy efficiency (%)	SmartChargeNet vs DCR	10 / 10	5.2	25.68	17.2	3.83e-15	1.23e-13	[4.8, 5.6]	11.5
Grid load variance (kW2)	SmartChargeNet vs SSP	10 / 10	-0.173	-53.14	14.1	1.28e-17	4.08e-16	[-0.180, -0.166]	23.8
Grid load variance (kW2)	SmartChargeNet vs DTAR	10 / 10	-0.157	-52.63	15.1	1.58e-18	5.06e-17	[-0.163, -0.151]	23.5
Grid load variance (kW2)	SmartChargeNet vs CMOO	10 / 10	-0.140	-46.93	15.1	8.84e-18	2.83e-16	[-0.146, -0.134]	21.0
Grid load variance (kW2)	SmartChargeNet vs RLR	10 / 10	-0.128	-47.05	16.3	7.80e-19	2.50e-17	[-0.134, -0.122]	21.0
Grid load variance (kW2)	SmartChargeNet vs MACC	10 / 10	-0.113	-41.54	16.3	5.85e-18	1.87e-16	[-0.119, -0.107]	18.6
Grid load variance (kW2)	SmartChargeNet vs CAGO	10 / 10	-0.100	-36.76	16.3	4.19e-17	1.34e-15	[-0.106, -0.094]	16.4
Grid load variance (kW2)	SmartChargeNet vs ETMG	10 / 10	-0.087	-35.23	17.4	1.20e-17	3.84e-16	[-0.092, -0.082]	15.8
Grid load variance (kW2)	SmartChargeNet vs DCR	10 / 10	-0.074	-29.96	17.4	1.93e-16	6.17e-15	[-0.079, -0.069]	13.4

Counts are totals over the held-out test episodes (episodes 1701 to 2000) averaged over 10 runs and rounded to the nearest session. Rated efficiency is the session-weighted connector-to-battery efficiency 0.90·fL2 + 0.85·(1 − fL2); the reported energy efficiency (Table 4) is lower than the rated value by the CC–CV taper-phase overhead described in Section 4.1.

Table S5. Fleet-level charging session counts by connector type on the SUMO-Urban-Grid test split.

Method	Total sessions	Level-2 sessions (7.2 kW)	DC-fast sessions (50 kW)	Level-2 fraction fL2 (%)	Rated efficiency (%)	Reported energy efficiency (%)
SSP	41,238	8,660	32,578	21	86.1	69.4
DTAR	40,917	9,411	31,506	23	86.2	70.9
CMOO	40,462	13,757	26,705	34	86.7	72.1
RLR	40,285	11,683	28,602	29	86.4	73.5
MACC	39,871	15,151	24,720	38	86.9	74.8
CAGO	39,604	16,238	23,366	41	87.1	76.2
ETMG	39,352	17,315	22,037	44	87.2	77.6
DCR	39,118	10,171	28,947	26	86.3	78.9
SmartChargeNet	38,274	23,347	14,927	61	88.0	84.1

Section S1. Synthetic sensor variable generation for NYC-EV-2024: equations, parameters, and seeds.

Scheme 1. 1 Vehicle classes and physical parameters. Each confirmed EV plate is assigned to one of three classes (Table S1.1). Parameters are taken from manufacturer specifications and published discharge curves and are held fixed per class.

Parameter	Tesla Model 3	Chevrolet Bolt	Nissan Leaf
Fleet share (%)	40	35	25
Usable battery capacity (kWh)	75	65	40
Nominal pack voltage (V)	350	350	350
Vehicle mass m incl. driver (kg)	1920	1690	1640
Drag coefficient Cd	0.23	0.31	0.28
Frontal area A (m2)	2.22	2.42	2.27
Rolling resistance μ	0.010	0.011	0.011
Internal resistance R0 (mΩ)	42	55	68
Polarization resistance R1 (mΩ)	18	24	31
Polarization capacitance C1 (kF)	24	20	16
Thermal resistance Rth (K/W)	2.5	2.5	2.5
Drivetrain efficiency ηdrivetrain	0.90	0.90	0.90

S1.2 Per-segment energy consumption. For each road segment of distance d with mean speed v, mean acceleration a, and grade θ from SRTM elevation, Eseg = [m·a·d + μ·m·g·d + 0.5·ϱ·Cd·A·v2·d + m·g·sin(θ)·d] / ηdrivetrain, with g = 9.81 m/s2 and ϱ = 1.225 kg/m3. Regenerative braking returns 15% of the kinetic energy dissipated on deceleration events; recovered energy is logged separately and not counted toward the energy

efficiency metric. Auxiliary load Paux is drawn once per trip from the uniform distribution U(0.3, 1.2) kW and added as Paux·Δt per 30 s epoch.

S1.3 Battery state (Thevenin equivalent circuit). Pack current at each 30 s step is I(t) = Pdemand(t)/Vterm(t), where Vterm(t) = VOC(SoC) − I(t)·R0 − V1(t) and the polarization voltage evolves as V1(t+Δt) = V1(t)·exp(−Δt/(R1·C1)) + I(t)·R1·(1 − exp(−Δt/(R1·C1))). State of charge is integrated as SoC(t+Δt) = SoC(t) − I(t)·Δt/(3600·Q), with Q the pack capacity in Ah. VOC(SoC) is a piecewise-linear lookup fitted to the published discharge curve of each class at eleven SoC breakpoints (0.0 to 1.0 in steps of 0.1). Initial SoC at trip start is drawn from U(0.2, 0.9). Charging follows the CC–CV rule of Section 4.1: connector rated power until SoC = 0.8, then power tapering linearly to zero at SoC = 1.0.

S1.4 State of health. SoH is initialized per vehicle from U(0.86, 1.00) and decremented by 2.4 × 10−5 per equivalent full cycle, with an additional calendar term of 1.1 × 10−6 per hour scaled by exp(0.035·(Tcell − 25 °C)). SoH scales the usable capacity Q and increases R0 by (1 − SoH)·0.6·R0.

S1.5 Cell temperature (lumped electrothermal model). Tcell(t+Δt) = Tcell(t) + (Δt/Cth)·[I(t)2·R0 − (Tcell(t) − Tamb(t))/Rth], with thermal capacitance Cth = 4.2 × 104 J/K per pack, Rth = 2.5 K/W, and Tamb(t) the NOAA hourly ambient temperature for the trip timestamp and TLC zone, linearly interpolated to 30 s.

S1.6 Charging-station occupancy. All generated trajectories are replayed against the 1200 AFDC station locations. Each station has four connectors (two Level-2 at 7.2 kW, two DC-fast at 50 kW) served by a FIFO queue; a vehicle requests charging when SoC < 0.2 and is routed to a station by the policy under evaluation. Session duration follows the CC–CV curve from the arrival SoC. Occupancy per 30 s epoch is the fraction of the epoch during which each connector was engaged. Connector-to-battery efficiency is 0.90 for Level-2 and 0.85 for DC-fast sessions.

S1.7 Sensor noise and missingness. Gaussian measurement noise is added to each synthetic reading before transmission: SoC σ = 0.5%, SoH σ = 0.3%, cell temperature σ = 0.4 °C, speed σ = 0.3 m/s, connector voltage σ = 1.5 V, connector current σ = 0.8 A, ambient temperature σ = 0.5 °C. Packet loss is simulated as an independent Bernoulli process with probability 0.02 per sensor vector per epoch; lost readings are handled by the imputation and missingness-flag rule of Section 4.1.

S1.8 Random seeds. The NYC-EV-2024 generation pipeline uses NumPy Generator seeds 20240101 (vehicle class assignment and initial SoC), 20240102 (auxiliary load and SoH initialization), 20240103 (sensor noise), and 20240104 (packet loss). SUMO-Urban-Grid scenarios use SUMO seeds 1 to 10 for the ten independent runs; PPO training uses PyTorch seeds 0 to 9 matched to the same run index. All seeds are fixed in the configuration files accompanying the dataset deposit.

Section S2. Reproducible EV plate linking procedure (Section 5.1).

The following Python listing implements Steps 1 to 4 of Section 5.1. Input files are the NYC TLC For-Hire Vehicle trip records (January to June 2024), the TLC Active Vehicle Database, and the EPA Green Vehicle Guide export, all downloaded from the sources cited in the main text. The output file NYC_EV_2024_plate_class_map.csv (2847 rows) stores the SHA-256 hash of each confirmed plate, its vehicle class, and its battery capacity.

```
import pandas as pd, hashlib

trips = pd.read_parquet("fhv_tripdata_2024_01_to_06.parquet")          # Step 1

active = pd.read_csv("tlc_active_vehicle_database.csv")

epa = pd.read_csv("epa_green_vehicle_guide_ev.csv")                     # EV rows only
```

```
norm = lambda s: s.astype(str).str.lower().str.replace(r"\s+", "", regex=True)
active["key"] = norm(active["Vehicle Make"]) + "|" + norm(active["Vehicle Model"])
epa["key"] = norm(epa["Make"]) + "|" + norm(epa["Model"])
plates = active[active["Fuel Type"].str.upper() == "ELECTRIC"]          # Step 2
plates = plates[plates["License Plate"].isin(trips["plate"].unique())]
plates = plates[plates["key"].isin(epa["key"])]                          # Step 3
class_map = {"tesla|model3": ("Tesla Model 3", 75),
             "chevrolet|bolt": ("Chevrolet Bolt", 65),
             "nissan|leaf": ("Nissan Leaf", 40)}                         # Step 4
plates = plates[plates["key"].isin(class_map)]
out = pd.DataFrame({
    "plate_sha256": plates["License Plate"].map(lambda p: hashlib.sha256(p.encode()).hexdigest()),
    "vehicle_class": plates["key"].map(lambda k: class_map[k][0]),
    "battery_kwh": plates["key"].map(lambda k: class_map[k][1])})
out.drop_duplicates("plate_sha256").to_csv("NYC_EV_2024_plate_class_map.csv", index=False)
```

Class counts in the output file: Tesla Model 3, 1139 plates; Chevrolet Bolt, 996 plates; Nissan Leaf, 712 plates; total 2847.

Section S3. Supplementary data file RewardLog_AllSeeds.csv.

The file contains 20,000 rows (10 seeds × 2000 episodes) with columns seed (0 to 9), episode (1 to 2000), and mean_reward, the unsmoothed mean per-step reward of Equation (6) over all decision steps of that episode. The curve in Figure 3 of the main text is obtained by averaging across seeds per episode and applying a 50-episode exponential moving average; the shaded band is one standard deviation across seeds.
